# The Good Life with Dementia approach: A realist-informed qualitative study of a peer-tutored course, co-produced with and for people living with dementia

**DOI:** 10.1371/journal.pone.0349444

**Published:** 2026-06-12

**Authors:** Kate Gridley, Mark Wilberforce, Rachel Mann, Laura Tucker, Beth Casey, Chris Clarke, Sam Creavin, Irene Donaldson, Howard Gordon, Damian Murphy, Mark Pearson, Mohammed Akhlak Rauf, Yvonne Birks

**Affiliations:** 1 Older Adults’ Social Care Research (OSCAR) Group, University of York, York, North Yorkshire, England; 2 Mental Health and Social Care Research Centre, School for Business and Society, University of York, York, North Yorkshire, England; 3 Tees, Esk and Wear Valleys NHS Foundation Trust, York, North Yorkshire, England; 4 Centre for Academic Primary Care, Bristol Medical School, Bristol, England; 5 Striving Towards a New Day (STAND), Fife, Scotland; 6 Deepness Dementia Media, Port of Ness, Isle of Lewis, Scotland; 7 Innovations in Dementia CiC, Exeter, Devon, England; 8 Wolfson Palliative Care Research Centre, Hull York Medical School, University of Hull, East Riding of Yorkshire, England; 9 Meri Yaadain CiC., Bradford, West Yorkshire, England; University of New Brunswick, CANADA

## Abstract

People with dementia often report a lack of post-diagnostic support, and much of the current dementia training available is for staff or carers, not for the person diagnosed. The Good Life with Dementia course was designed with and for people with a diagnosis of dementia and is co-delivered by peer-tutors living with dementia, supported by a trained facilitator. This study used realist-informed methods, underpinned by a co-productive ethos which values all sources of expertise equally, to better understand the core constructs underpinning the Good Life approach and how these operate to produce outcomes. The resultant, evidence-based programme theory suggests that – in a context characterised by shared experience, equality and positive expectations – three key mechanisms can trigger: sharing of experiences and resources; peer-led learning and responding; and the taking on of meaningful roles. Qualitative evidence indicates that these mechanisms are likely to lead to four interconnected outcomes: enjoyment; feeling valued (personhood); (re)building social confidence and connections; and positive reframing of life with dementia, meaning participants felt more prepared to face the challenges ahead. Not everyone diagnosed with dementia will want to take part in a peer-led course, but interventions like a Good Life with Dementia could be part of a suite of post-diagnostic options available to help people with dementia to live as well as possible. The next step will be to establish whether the approach can be manualised, delivered with different communities and evaluated in trial conditions. This will be assessed via an inclusive feasibility study already underway and due to conclude in August 2027.

## Background and introduction

Receiving a dementia diagnosis is a significant life event [1–4] and transitioning to life after diagnosis can involve uncertainty and loss [5–8]. Post-diagnostic interventions aim to support individuals to feel they have a future with possibilities and purpose [9,10]. However, research has repeatedly highlighted gaps in such provision, suggesting a need to improve the range of post-diagnostic options available and ensure they meet the needs of people from all backgrounds who find themselves living with dementia [5,6,11–14].

The dementia strategy for England identified peer-support as a promising approach to ensuring people with dementia receive the practical and emotional support they require after diagnosis, calling for more research to underpin an evidence-based approach [15]. There is a substantial literature on peer-support for other populations [16–20] but less is known about the defining features or effectiveness of peer-support by and for people with dementia, despite pockets of innovative practice (see for example the DEEP network in the UK). Price et al. [21] mapped the international evidence on peer-support, identifying 91 systematic reviews, randomised controlled trials and economic evaluations of peer-support, but found no studies of this nature looking at peer-support for people living with dementia. Whilst qualitative research suggests benefits from dementia peer-groups include increased confidence, sense of belonging and ability to manage daily life [22–26], evidence on the core features of effective dementia peer-support is absent and there remains therefore a lack of clarity regarding the mechanisms operating in such models or how these function in different contexts, for whom and to what end. Lack of robust evidence in this area may prevent routine commissioning of peer-to-peer dementia interventions, and mean health and social care professionals are hesitant to refer into related services [27,28].

This paper addresses this evidence gap with a specific focus on the Good Life with Dementia course, a peer-led post-diagnostic course co-produced by people living with dementia and co-delivered by them. Whilst there are numerous courses designed to support and inform the carers of people living with dementia [29] and some for both people living with dementia and their families/carers [30,31] the Good Life approach is novel in its emphasis on peer-to-peer support exclusively for people with a diagnosis of dementia themselves.

### Peer-support: Theory and evidence

Peer-support is an established feature of post-diagnostic support for people with some health conditions (notably cancer, HIV, diabetes and mental illness) although the evidence on effectiveness remains mixed [16–20]. Theoretically, peer-support has the potential to be transformative for people living with any long-term condition. In the mental health context, Mead et al. [32] theorised that a sense of understanding based on mutual experience in peer-support builds trust and validation, creating a culture of ability leading to improved wellbeing. Reviewing the literature across multiple types of peer-work, Watson et al. [33] identified ‘love labour’, a focus on strengths and the ‘helper role’ of peers who occupy a liminal position (between services and lived experience) as key mechanisms present in peer-support. Sullivan et al. [34] noted that, in the context of dementia, literature on peer-support tends to draw on Kitwood’s theory of personhood [35], ideas of citizenship [36] and the social model of disability [37]. Important features are, for example, interaction ‘in a safe space’ ([27], p 247) and an absence of hierarchy leading to a sense of ‘significance’ (feeling valued and valuable) [38] which may be particularly meaningful for people with dementia who can, in other contexts, be invalidated [35].

Regarding the specific features of peer-support, Sullivan et al. found that hospital-based peer-support models tended to be time-limited, professionally designed and focussed on diagnosis-specific psychoeducation. Community models, in contrast, are often less professionally directed. The degree to which dementia peer-support groups require professional support is a salient question, given that a defining characteristic of dementia is cognitive impairment including problems with memory, communication and executive function and so people with the condition may require more support to arrange and deliver sessions than people with other long-term conditions.

Recently, there have been moves to apply the principles of recovery (first developed in mental health) to dementia care [39] where recovery is conceptualised as ‘helping people to rebuild lives that they find satisfying, meaningful and valued’ ([40], p2). A small number of co-produced, peer-led Recovery College dementia courses are available in the UK [41]. However, these courses tend to be for staff and family carers as well as people with dementia [30,42] and it remains unusual to find co-produced recovery focussed post-diagnostic courses provided exclusively for people living with a diagnosis of dementia.

### The Good Life with Dementia course

The Good Life with Dementia course was originally designed by a group of people living with dementia, with support from a third sector organisation specialising in dementia empowerment and engagement (http://www.innovationsindementia.org.uk/). The course has since been delivered in several areas of the UK, usually hosted by a local community provider, often with the involvement of local health and social care professionals. However, there are no fixed referral routes into the Good Life course as it is not yet an established part of usual dementia care.

The course consists of six or seven weekly group sessions of approximately two hours (in person) delivered jointly by peer-tutors, with as much or as little support as they need from the trained facilitator. Whilst retaining this basic structure, each new iteration of the course is co-produced by peer-tutors (people living with dementia) who agree a programme for the course they will co-deliver. The aim is to provide an opportunity for people with expertise of living with dementia to support others more recently diagnosed to adjust to living with the condition. How this is done will differ by group, but in common across all courses is the chance for people with dementia to:

- discuss what living with dementia means to them
- learn and share amongst equals
- explore new ways of ‘living as well as possible’ with dementia
- find out which services might be available to provide further support

Three co-production meetings are held with peer-tutors and the facilitator in advance of each course to build the course programme, typically starting with the question: *what message would you like to give someone after a diagnosis of dementia, based on your own experiences of going through this?* The facilitator provides practical help to put the course together (booking speakers if required, and a venue) as well as tailored support and encouragement to scaffold the peer-tutors’ co-delivery of each session. Facilitators, and the organisations they work for, also have a role in managing potential safeguarding issues or distress, through the application of their organisation’s existing policies and procedures.

Learners on the course are people diagnosed with dementia signposted through local memory, social care and voluntary sector services. Family carers are not encouraged to attend the course sessions but may congregate in an adjacent room, and are given written information about topics covered and key resources in a letter after each session (jointly addressed to the person living with dementia and any family or other supporters they identify). Towards the end of the course the group jointly considers next steps, which may require input from the facilitator and/or their organisation (for example, the creation of an ongoing peer-support group). A potential next step is for learners on one course to become tutors on the next.

### The Good Life research

The realist-informed study reported here, grounded within the Medical Research Council framework on evaluating complex interventions [43], was designed to test an initial stakeholder-generated theory of change (S1 Fig) to establish a model for subsequent manualisation and robust evaluation of the Good Life approach. Whilst theory of change and realist evaluation are distinct approaches to understanding complex causal processes, they can fruitfully be used together to examine these at different points of intervention development [44]. We aimed to move from the initial theory of change to a more developed programme theory by comparing elements of the initial theory against qualitative evidence drawn from two iterations of the Good Life course. Our objectives were to assess if and how the proposed mechanisms in the initial theory operate in practice, and whether and for whom they lead to proposed outcomes. Other elements of the study included gathering insights from people with experience of dementia from South Asian communities to learn about the potential transferability of this model to different community contexts. Findings from this latter work are reported separately.

## Methodology and methods

We used methods informed by realist ontology, which asserts that hidden causal forces (mechanisms) underpin the outcomes we observe from interventions (or programmes) [45]. These mechanisms can remain latent until activated in the right conditions (contexts) to produce the outcomes we measure. This approach was selected as it lends itself not only to studying *if* an intervention works, but *how* it could work for different people in different contexts or communities, and under what circumstances it might not work for them. Epistemologically, realists argue for a cumulative approach to understanding programmes, gathering evidence on underpinning mechanisms (which will always be partial because these mechanisms cannot be directly measured) from multiple sources [45]. Qualitative methods are well suited to this cumulative approach, and we used a combination of qualitative interviews, group discussion and observation, informed at all stages by lived experience.

The study was approached with the same co-productive ethos as the intervention itself, with stakeholders involved in all elements of the research, from design through to dissemination. Advisors living with dementia also worked as co-researchers contributing to data collection and analysis. Realist-informed studies have traditionally not employed co-researchers [44], but the involvement of people with dementia in research as equal partners is increasingly being recognised as important for equity and quality [46,47] and these principles are consistent with the cumulative realist approach which draws on the full range of available sources of evidence.

The research was funded by the National Institute for Health Research (NIHR) Three Schools Dementia Research Programme. Ethical approval was granted by the West Midlands – Coventry & Warwickshire Research Ethics Committee on 22^nd^ May 2023 (REC reference: 23/WM/0088; IRAS project ID: 324935).

Recruitment and consent: This paper reports findings from observations of two Good Life courses and qualitative interviews with participants (tutors and learners) living with dementia on these courses, supplemented by group discussion. Both courses were facilitated by Innovations in Dementia CiC in the north of England in summer and autumn 2023. Participants were recruited between 5^th^ June 2023 and 31^st^ January 2024. All of the people with dementia participating in the courses, as peer-tutors or as learners, were eligible to take part in the research, providing they had capacity to give informed consent. Potential participants were informed in person about the research in advance of the course by the facilitator (DM, who was a co-applicant on the project), using easy read information sheets with visual prompts. [48] (S2 File). Full information and a further opportunity to ask questions was given by a university researcher at the first observed session, with support from a co-researcher with lived experience. It was stressed that learners and tutors could take part in the course without participating in the research. Those who chose to join the research signed a written consent form or gave verbal consent witnessed by a researcher. In keeping with the Mental Capacity Act (MCA) [49] mental capacity to give informed consent was assumed unless there was reason to doubt this. If in doubt, the researcher referred to four key questions specified in the MCA to assess capacity (S3 File). Informed consent was treated as an ongoing process and confirmed at each session via verbal reminders, regular opportunities to ask questions about the research, and visual cues (S4 File). “I want to speak” cards were issued to all course attendees to enable them to speak up at any time (for example if they changed their mind about being observed) without having to verbally interrupt an ongoing discussion (S5 File).

Data collection: The first observed Good Life course (GL1) ran from May to July 2023. This was a 7-week course and researchers observed all but the first two sessions (which were missed because recruitment could not commence until ethical approval was received on 22^nd^ May). Observation of the remaining five sessions equated to 450 minutes of observation. Two university researchers (RM, a health services researcher and KG, a dementia care researcher) and one co-researcher living with dementia (WM, a dementia rights campaigner with a health service background) were involved in these observations. GL1 was attended by 12 learners and two tutors living with dementia, all of whom gave informed consent for the observations and qualitative data collection. Six learners and both tutors on GL1 took part in individual, realist-informed interviews to help develop the programme theory. All 14 participants joined a group discussion in the final course session about their experiences of the course, which was facilitated by the course facilitator.

GL2 was a 6-week course which ran from October to November 2023 in a different location from GL1 with different participants and tutors, but the same facilitator. All six GL2 sessions were observed (equates to 540 minutes of observation) by one of two university researchers (RM and LT, a researcher with experience as an adult social care practitioner), and a co-researcher living with dementia observed two sessions (HG, who had experience of being a participant on an earlier Good Life course). GL2 was attended by seven learners and one tutor, all of whom gave informed consent for the observations and qualitative data collection. Five learners and the peer-tutor living with dementia took part in individual, realist-informed interviews to help develop the programme theory. In the final session a researcher (RM) facilitated a focus group discussion with all participants following a topic guide (S7 File. Topic guide for focus group).

For both courses, observation consisted of audio-recording and making written notes on context, atmosphere and the interactions between learners, peer-tutors and the facilitator. Academic researchers completed an observation template with free text boxes (S8 File. Observation record – blank) and co-researchers wrote unstructured notes (in accordance with their preferences). Interviews combined realist interviewing techniques [50] and the principles of inclusive dementia research [47,51]. In realist interviewing, questions grounded in the initial programme theory are asked to prompt exploration of the different elements of the initial theory. Topics are selected in advance, linked to the objectives of the study and the hypotheses being tested, but the questions asked can be tailored to fit the context of individual interviews and the likely expertise of different interviewees [50]. Thus, different questions could be asked of course learners and course tutors, but all with the aim of testing elements of the initial, stakeholder-generated theory of change and refine understanding of the core elements underpinning the Good Life model. Our approach to inclusion was informed by calls for more flexibility in the field, combined with the meaningful involvement of people with dementia in study design, to ensure a better ‘fit’ between research practices and participants’ preferences and realities [47]. All interviews were held in the community setting known to participants at a time convenient to them. Duration ranged from short (micro-) interviews in session breaks, to full length (up to 52 mins) interviews depending on the preference of participants. Visual aids, developed with co-researcher input, were used to introduce different elements of the initial theory of change in a user-friendly format (S6 File). Interviewees were asked to what extent these hypotheses reflected their experiences and whether there were other things that should be considered. The following is an example of this approach to questioning, taken verbatim from the transcript of the interview with a learner in GL1:

Q: *So one of the things that was highlighted in the work we did before was about hope. Do you feel like your feelings of hope about dementia, about your condition, have changed since you’ve been on the course?*

Most participants were interviewed immediately after a Good Life session (or even during a break) to maximise recall. Where it was not possible for a participant to be interviewed on the day, the researcher met with them soon after (ideally within a few days, but two interviews were conducted nearly two months after, both times on the request of the participant).

### Analysis

To ensure rigour in analysis we followed the four realist fundamentals of (1) simultaneous data collection and analysis, (2) retroductive theorising, (3) configurational analysis and (4) realist analysis quality [52]. Data collection and initial analysis were undertaken concurrently (fundamental 1) to enable iterative testing and refinement of the programme theory (fundamental 2) following the three stages of theory gleaning, theory refinement and theory consolidation [50], see Table 1. An adapted realist-informed version of the Framework approach [53] was developed and used by four researchers (KG, RM, LT and BC) who met regularly to discuss and agree interpretations. Column headings reflected context/mechanism/outcome configurations (fundamental 3) derived from the initial (pre-research) stakeholder theory of change, supplemented by potential mechanisms of peer-support identified in the literature. Analysis then proceeded abductively [54], with researchers charting both to these theory-derived headings whilst also developing new candidate mechanisms inductively from the primary data (noting relationships to context and outcomes) where these differed from the initial theory. We interpreted fundamental 4 (realist quality) as consisting of trustworthiness, coherence and plausibility. Trustworthiness was promoted through our iterative approach to theory building and testing, and the meaningful involvement of people with lived experience in all stages of this. Coherence and plausibility were further promoted in the theory refinement and consolidation stages where candidate elements of the theory were presented to those with expertise for comment and further developed. This approach involved the wider advisory group (consisting of dementia care professionals, people with lived experience of dementia and researchers) to sense check and verify components of the emerging theory. Theory consolidation began with a two-day analysis workshop with this group where members, including co-researchers living with dementia, worked from sections of transcript to refine our emerging understanding. This was followed by two knowledge exchange events where the proposed programme theory was presented, and views incorporate from a wider group of stakeholders.

**Table 1:** Three phases of theory development.

Stage	Description	Good life research
1: Theory gleaning	Data collection and initial analysis to help identify how circumstances (C) may impact behaviour (M) and effectiveness (O)	Observations of GL1 and interviews with GL1 learners and tutors Initial analysis (CMO charting using Framework and discussions with wider Advisory Group members, including experts by experience)
2: Theory refinement	Refine understandings of specific outcome patterns (CMO configurations) Data collection at this stage is more tailor-made to test interpretations from the first stage of analysis	Observations and more tailored interviews with GL2 learners and tutor. Question example: “*When we spoke to learners on previous courses they talked about the importance of ‘shared experience’ – is this something that’s important to you? If so, what does shared experience make possible?”* Further analysis (CMO charting using Framework and discussions with Advisory Group members, including experts by experience)
3: Theory consolidation	This phase gives more detailed consideration to a smaller number of CMOs	A two-day residential workshop held with Advisory Group members, including experts by experience, looking at raw data and candidate CMO configurations, to further refine the theory. Two final stakeholder workshops with wider stakeholders (people with lived experience, service providers and commissioners) to share findings, hear feedback and consolidate the final theory.

### Findings

The findings presented below relate to interview, observation and group discussion data from 22 people living with dementia who participated on the Good Life courses (14 on the first course, eight on the second).

### Participants

All of the 22 Good Life course participants across the two observed courses chose to take part in an end of course discussion, and 14 of these also took part in a qualitative interview either during the course or soon afterwards. An equal spread of men and women took part in these interviews, aged from 61 to 81, but there was no ethnic diversity in the sample. Other key characteristics of interview participants are given in Table 2.

**Table 2:** Interviews with participants with dementia.

Interview ID*	Course	Learner or tutor	Age	Living circumstance	Type of dementia
**A**	GL1	Learner	75-84	With spouse	Unknown**
**B**	GL1	Learner	75-84	With spouse	Vascular
**C**	GL1	Learner	65-74-	With spouse	Unknown
**D**	GL1	Learner	Under 65	With spouse	Mixed
**E**	GL1	Learner	65-74	With spouse	Alzheimer’s
**F**	GL1	Learner	65-74	With spouse	PCA***
**G**	GL1	Tutor	Under 65	Lives alone	FTD****
**H**	GL1	Tutor	75-84	With spouse	Unknown
**I**	GL1	Learner	75-84	With spouse	FTD
**J**	GL2	Learner	65-74	With spouse	Unknown
**K**	GL2	Learner	75-84	Lives alone	Vascular
**L**	GL2	Tutor	65-74	With spouse	Unknown
**M**	GL2	Learner	75-84	With spouse	Alzheimer’s
**N**	GL2	Learner	75-84	Lives alone	Alzheimer’s

* To protect anonymity, tutors are not referred to by Interview ID in this paper but as Tutors 1, 2 or 3

** Unknown indicates that the person with dementia did not know the type of dementia

***Posterior cortical atrophy

****Frontotemporal dementia

### Structure of findings

The aim of this study was to refine our understanding of the mechanisms through which the outcomes of a Good Life course are achieved and under what circumstances participants may not see these outcomes. We also developed our understanding of the outcomes themselves, using empirical data from observations of group interactions as well as the expressed views of participants about their experience of the course and its consequences. This section starts by setting out this refined understanding of the potential outcomes of the Good Life course, before moving on to consider the mechanisms (things happening) which may have led to outcomes, the context underpinning this, and the situations in which outcomes may not be achieved.

### Theorised outcomes

The original (pre-research) theory of change, devised by stakeholders in advance of this research (S1 Fig Initial [pre-research] theory of change), identified possible outcomes of the Good Life course for people with dementia including increased knowledge about living with dementia, a sense of confidence and empowerment, a sense of connection and community, and availability/access to community resources (including a long-term peer-support, self-advocacy group). It was suggested that these interim outcomes could lead to the overall outcomes of ‘subjective wellbeing’ and ‘hope’.

### Observed outcomes

**Fig 1:**
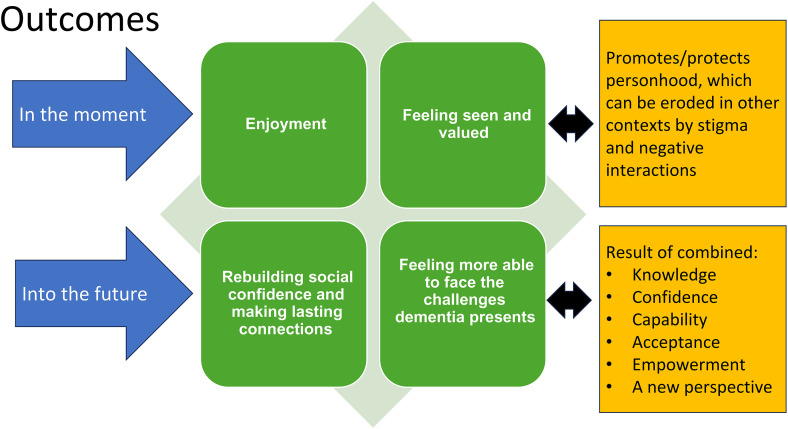
Outcomes identified through realist-informed qualitative study.

Our findings were broadly supportive of these theorised outcomes, but our collaborative analysis offered some refinements. Specifically, outcomes including increased knowledge, confidence and access to community resources (which were confirmed through our realist-informed interviews) culminated in an overall sense of feeling more able to face the challenges dementia presents. Participants also reported improved (rebuilt) social confidence, and some felt that they had gained lasting social connections that would continue as their dementia progressed. More proximal outcomes included simply having fun on the course, as well as the promotion of personhood (participants feeling seen and valued by others on the course) something that in different contexts could be lacking. Fig 1 sets out, visually, the outcomes framework derived from data analyses.

It should be noted that not everyone reported the full set of positive outcomes, and one participant in particular felt that the course had not delivered the outcomes she hoped for. Below we expand upon the outcomes identified, as well as the discordant views of this less satisfied participant, before moving on to consider potential mechanisms and context factors that can underpin outcomes.

### In the moment benefits

All the participants interviewed said that they enjoyed attending the course and had fun whilst they were there. These experiences of emotional well-being ‘in the moment’ held value in and of themselves. Moreover, it was noted that spending enjoyable time with other people living with dementia had helped participants to see their situation differently ‘…*it’s nothing to be ashamed of.’* (Learner B, GL1). A contrast was highlighted between the ways people in the group interacted on the course and how participants sometimes felt they were treated in other settings. Participants explained that outside of the group they did not always feel recognised, visible or valued as a person, using words like ‘*outcast’* and ‘*floating about’* or, as this participant said:

*I have found that you can go places and you’re ‘not in the room’… Well, that makes me sad.* Learner I, GL1

Relaxing and having fun with others who treated them as equals helped to reinforce participants’ sense of personhood, which in other contexts they felt had been eroded since the onset of dementia.

### Longer lasting benefits

Several participants commented that they had made friends on the course and hoped to stay in touch with them afterwards. Some made a direct connection between these new social contacts and their future resilience, as this participant explained: ‘*if I want anything, they’re there for me.’* (Learner C, GL1). Others reported a growing social confidence that could be applied outside the immediate group: ‘*I think I just fit into company now*’ (Learner M, GL2). Some participants located the course within a narrative of having lost and regained social confidence, ascribing the Good Life a key a role in this story of social recovery:

*At one time I didn’t like to speak about anything because I got a little bit of embarrassment about my own mind going haywire and losing it, and then I just built back up and got my confidence back.* [The course] *has helped, and it’s just fed me as well, and it’s just given me more confidence to be able to communicate with everybody that’s around.* Tutor 1

Alongside this rebuilt social confidence, participants noted a changed perspective about their future with dementia. The original (pre-research) theory of change characterised this as ‘increased hope’. Our interviews confirmed that participants felt they had increased knowledge, new skills and access to more relevant resources, which contributed to them feeling more able to face the future with dementia. However, when asked specifically about ‘hope’ participants were more equivocal, linking hope with the biomedical progression of their dementia which they knew was not going to improve. This nuance is demonstrated in the following interview excerpt:

*Q: Do you feel like your feelings of hope about dementia, about your condition, have changed since you’ve been on the course?*

*L: Hope, I mean, you don’t feel that you’re going to get better at the end of the day because we’re not but when you see people, and everybody’s getting together, and it is cheerful, and that helps a lot I think.* Learner F, GL1

Instead of ‘hope’ per se, participants talked about having an altered perspective on their future, now that they had more knowledge and people to share their concerns with: ‘*It’s stopped me being scared.’* (GL2 focus group). Our refined programme theory therefore talks not of ‘hope’ as an outcome, but of the potential for people to feel more able to face the challenges dementia presents. In the words of one peer-tutor:

*‘…come on those courses and you won’t be scared for much longer. You’ll know what it’s all about, and you’ll have a different attitude towards it and take a different approach with it.’* Tutor 2

### Discordant views

There was one participant (on GL2) whose experience stood out because she felt the course had not met her expectations. This participant placed low value on group interaction, describing herself as ‘*an island’* and commenting *‘It’s never something that I was keen on, banding together with other people*.’ When asked about the outcomes of attending the course she said that it would not change the trajectory of her disease, it’s more ‘*a sort of social shoring-up exercise’* (Learner N, GL2). However, she continued:

*On the other hand, when something like this comes along, which I have never considered … I will go along and see if anybody else has got things that they do that help, so I could do to help as well…* [and] *it does seem that there is a wide range of problems that people are having, which are the same as mine…* Learner N, GL2

In the absence of a more well-matched source of support, therefore, it appeared that this participant did derive benefit from meeting people who were experiencing similar challenges to her and learning from the strategies they shared. It is worth noting, however, that the group was not her preferred mode of service delivery and for every Learner N who attended the course with reservations, there may have been several more who chose not to attend because a group-based peer-led course did not appeal.

### Mechanisms leading to change

Taking the ‘Essential Ingredients’ and ‘Key assumptions’ identified in the original theory of change as starting points, interviews with participants explored whether these, or other factors, were instrumental in leading to the outcomes they reported. Findings from early interviews were viewed within the wider context of observational data from GL1, and in the GL2 interviews and observations the theory was refined. Through this process, three key mechanisms were agreed to be likely to contribute to beneficial outcomes, each of which is considered below:

- people with dementia sharing their experiences, knowledge and resources
- engaging in peer-led learning and responding
- taking on meaningful roles

### Sharing experiences, knowledge and resources

Participants told us that the non-judgemental atmosphere and genuine interest borne of common experience on the course (in particular, learners and tutors all having a diagnosis of dementia) helped them to feel safe enough to share their own experiences, in a way some would not (and in some cases could not) do in other contexts:

*’It was being able to talk to somebody who, first of all, was interested, and took it and also took it on board. You couldn’t do that if you were in a pub having a drink, and you’d try to explain, well, I wouldn’t even bring the subject* [of living with dementia] *up, to be quite honest.’* Tutor 3

This sharing amongst interested peers helped participants to find connections between themselves and other group members and contributed to the sense of personhood identified above. It was noted that dementia and related concerns were not seen as acceptable topics of conversation in some contexts, even at home amongst family: ‘*Nobody talks about it*’ (Learner B, GL1). In contrast, being in a group with other people in similar circumstances enabled participants to talk openly about their dementia and feel less alone in their experience:

*L: You don’t feel as though it’s, “Why me? Why me?”* [Instead people say] *“Well, I’ve got it, and I’ve got it, and I’ve got it.”* [Not] *“Shut up, you miserable bugger.” (I get told off quite frequently.)*

*Q: So other people saying, “I’ve got it” that makes you feel…?*

*L: It’s more common-or-garden… Not just you.* Learner E, GL1

Participants also learnt valuable strategies through the sharing of experiences, resources and practical ways they had overcome challenges. Tangible examples included ideas for new hobbies, navigating the benefits system, and finding ways to exercise safely. Participants reported that this gave them confidence that they would be able to tackle future problems as they arose:

*It gives me more confidence within myself to actually deal with things, and not to feel embarrassed if I’ve got to ask someone else, ‘What do you think I should do about this?’* Tutor 1

### A peer-led approach

The peer-led approach and the ways participants drew on lived experience to respond to each other’s questions and concerns was another important mechanism, and one which some participants had not encountered before:

*…this is the first time I’ve been able to sit down amongst a group of people that have got the condition in one way or another, and you listen to what they’re achieving or what they’re not achieving or what can be done, and I think that’s been very valuable…* FG, end of GL1

In more traditional health and social care settings, information and advice tended to come from professionals. Participants commented that professional-led interventions did not always help them to feel confident ‘*I get nervous with doctors*.’ (Learner D, GL1). It was suggested that information learned from others with dementia could be more relevant than that received in more traditional medical settings:

*It’s like going to the doctor, isn’t it? You’re telling him your symptoms. He’s replying to you with what he’s read in the book or been taught. Yes. He hasn’t a clue what it is* [to live with dementia]. *Even though he’s been a doctor for years.* Tutor 3

Several instances were recounted of participants being struck by how apt the responses of their peers were to the experiences and feelings they shared. From something as small as understanding that the return from a holiday can be disorientating, to as significant as demonstrating that life with dementia can still be worth living. A co-researcher with lived experience further noted, after observing sessions for the research, that not having professionals or family carers in the room enabled honest conversations and challenged ‘assumptions of incapability’:

*Excluding professionals/family/carers apart from maybe the facilitator from the group challenges those assumptions and enables honest conversations/topics between people living with dementia which otherwise wouldn’t take place*. Co-researcher living with dementia (email)

On the Good Life course, participants with dementia are encouraged to pursue their own agendas, from the initial co-production of the course to the peer-to-peer discussions at each session. As one participant commented: ‘*It wasn’t like a classroom. We weren’t …being taught.’* (Learner M, GL2). It was further suggested by an advisor with lived experience that people (in general) are more likely to take ownership of their own learning when they are trusted to do so. Interviewees echoed this observation, indicating that the peer-led approach, underpinned by positive expectations, helped them to realise that they had the capacity to help themselves:

*You know what to do to help yourself, you become a little bit independent then. Which takes the pressure off you, wondering whether you’re doing the right thing. It also takes the pressure off the people who are caring for you…* Tutor 3

### Taking on meaningful roles

The original (pre-research) theory of change formulated by stakeholders proposed that tutors on the Good Life course act as role models to people newly diagnosed with dementia. Our evidence suggests that tutors did indeed see themselves in this way and drew confidence from this role. In addition, group members newly diagnosed with dementia acted and could be viewed as role models themselves. One participant reflected on the significance to her of having met a group of other people (tutors *and* learners) in her position who were doing things she did not think a person with dementia could do:

*Now, I’ve seen all these other people here, and they’re doing, they’re not thinking of it as an illness. It’s more like, you know, er, a mental illness, but that doesn’t stop them doing any things.* Learner C, GL1

From observations it was clear that some people contributed in sessions more than others, and there were some who shared relatively little about themselves and their experiences. Nevertheless, interview findings from these participants tallied largely with the wider findings, indicating that active contribution may not be a prerequisite for deriving benefit from the course. This person, for example, spoke little during the sessions but said afterwards:

*I just find it so relaxing and there’s something about it. We’re all joined, just a joint thing like that.* Learner K, GL2

Simply attending and being a participant in the group could therefore offer a meaningful role, particularly if prior to this the person had been struggling to find purpose. Another participant who spoke relatively little during the sessions, (Learner D, GL1), for example commented that the group had given her a reason to get up in the morning.

We observed that group members could spark off each other and had fruitful conversations that required little input from the facilitator. However, the facilitator often played a key role in catalysing these conversations and supported peer-tutors to do the same. Creating the context within which such interactions could thrive took skill and preparation. Some structure and prepared content (a topic to discuss, a co-produced script for the tutors, or presentation from a relevant service) was required and the facilitator had to be ready to encourage and ask questions if participants (or peer-tutors) were struggling. Interviewees commented that the Good Life facilitator had a *‘wicked sense of humour’* which helped to create a relaxed atmosphere where people felt confident to chat and connect:

*‘This is the closest thing I’ve ever got back to a banter*.’ Learner E, GL1

Another part of the facilitator role was to create a trusting environment in which learners and tutors felt confident to manage difficult or emotive conversations amongst themselves, drawing on their own experiences and ability to empathise. In some situations, specific resources or information were required, and for this the role of the facilitator was essential.

### Context underpinning the course

Our findings suggest that the above mechanisms were operating within a context characterised by equality, shared experience and positive expectations. Participants described the atmosphere as friendly and trusting: somewhere they felt able to share their feelings and experiences without fear of judgement or consequence. This was also reflected in our observation notes:

…*conversations developed from one thing to another. Learners felt able to share and ask questions unhindered and relaxed*. Expert by experience notes from observation of GL1, session 3

Shared experience appeared key to this relaxed atmosphere: participants seemed confident that their perspective would be understood by those present because they were in similar circumstances and so could empathise. When connections were made, this also contributed to a sense of equality and camaraderie (several people described being ‘in the same boat’) and feeling less alone in their experience of dementia:

*…after you’ve got that first piece of paper that says the word dementia, and you think, oh heck, what’s this mean? Then being able to just be put with people in the same situation is so useful. That’s what I think. I think it was God sent that we got on that course.* Learner M, GL2

This seemed particularly important to participants in the wider context of a society where the dominant dementia discourse centres on individual failings. Finding connections with others in a similar situation shifted the locus of problems from individual deficit to common challenges:

*…you can look around this table and think we’re all feeling the same. You haven’t got people looking at you thinking what a right twit he is, because we understand how each other feel…* GL2 focus group

It was suggested that people with dementia could experience imbalances in interaction with people without dementia, who might speak over them or down to them. Participants felt they could trust their peers in the group not to express the sorts of negative opinions that they encountered in other contexts.

Our interpretation is that the positive expectations encountered on the course regarding both the possibility of living well with dementia, and the potential for all people with dementia to have something valuable to contribute, set the scene for the changed, more positive perspective observed. Serious subjects were discussed, but the facilitator’s approach was asset-based and participants with dementia were in a good position to respond empathetically to each other, informed as they were by shared lived experience.

### Summary of consolidated programme theory

**Fig 2:**
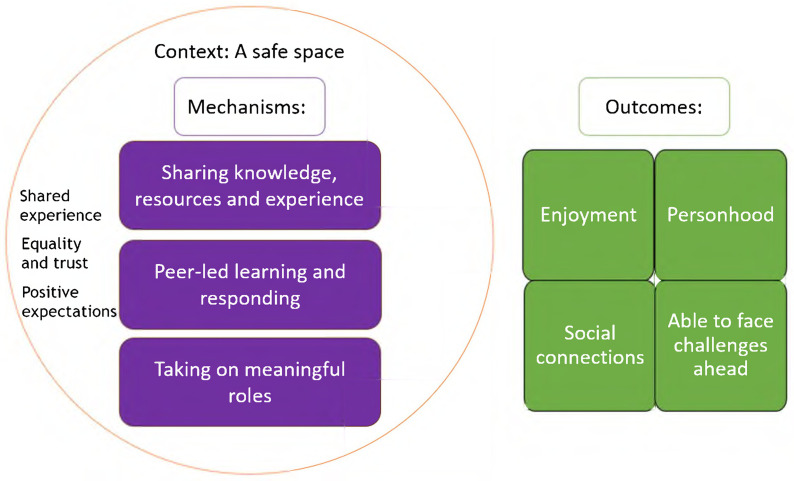
Good life with dementia programme theory.

In summary, the programme theory that was refined and consolidated through this research centres on the creation of a group context characterised by shared experience, equality and positive expectations (Fig 2). People with dementia told us they felt safe within this course context to share their experiences, knowledge and feelings without fear of judgement. Sharing in this way led to positive (qualitatively expressed) outcomes for most participants, including a renewed sense of personhood and a changed perspective on the challenges that can accompany living with a dementia diagnosis. Other mechanisms contributing to these outcomes were the peer-led nature of both the content and the delivery of the course; and the opportunities for all participants to take on meaningful roles.

The presence of divergent experiences, and in particular the views of one participant who felt the course did not meet her expectations, is evidence that these mechanisms will not necessarily activate for everyone.

## Discussion

This qualitative realist-informed evaluation set out to better understand the core elements of the Good Life with Dementia approach and determine how these might lead to outcomes for people living with dementia. The resultant, evidence-based programme theory asserts that – in a context characterised by shared experience, equality and positive expectations – three key mechanisms can be triggered: sharing of experiences and resources; peer-led learning and responding; and the taking on of meaningful roles. Qualitative evidence suggests that these mechanisms in context are likely to lead to four interconnected outcomes: enjoyment; feeling valued (personhood); (re)building social confidence and connections; and positive reframing of life with dementia, meaning participants felt more prepared to face the challenges ahead.

These findings tally with previous studies of social support for people living with dementia and memory loss which highlight that, through sharing experience and responding with empathy, peers can help each other to build connections and confidence [55]. Hagan and Campbell [24] highlight the potential role of peer groups in challenging negative stereotypes and rebuilding confidence, and we observed that the Good Life course achieved this partly by creating an environment where people treated each other as equals and felt mutually respected. The camaraderie that can come from spending time with people ‘in the same boat’ has been identified in other contexts, including MND [56] and mental health [57], challenging earlier theories suggesting that difference could be a key mechanism leading to benefits through peer-support. Whilst comparisons with others in a different situation may give hope or help to put difficulties in perspective, this research supports the theory that being with people who understand each other because they share lived experience can foster a sense of ‘common humanity’, supporting connection and mutuality.

A sense of ‘common humanity’ might be particularly important for people with dementia, as a population who have previously reported feeling they are ‘*no longer taken seriously in an equal, reciprocal relationship, and in some cases even felt they were no longer approached as a ‘complete human being*” [[7], p18). People with dementia can feel decentred and overlooked [1,35,58], with the locus of communication often shifting from the person with dementia to a caregiver, even during the early stages of the condition [59]. A core feature of the Good Life course is that it is specifically for people with a diagnosis of dementia (family carers are not in the room) *re*centring the person and enabling direct peer-to-peer support on an equal footing.

There are notable parallels between the potential outcomes of the Good Life approach and factors thought to contribute to overall social health for people living with dementia [60]. In particular, social connectivity and a sense of being more able to face the challenges dementia presents, relate to concepts like resilience (as both an outcome and a process of ongoing positive adjustment) which has been flagged as a measurable, strengths-based outcome important to people living with dementia [61]. Similarly, there are clear overlaps between the principles of recovery (rebuilding satisfying, valued lives [39,40]) and the Good Life model, but a difference in focus between existing Recovery College dementia courses (which tend to be for staff and family carers as well as people with dementia [30]) and Good Life where tutors and learners are all people with a dementia diagnosis.

The key mechanisms we identified underpinning the Good Life approach were not dissimilar to those noted in other forms of peer-support [32,33,62]. They correspond with Mead’s [32] theory that peers connect over a sense of shared understanding which is validating and creates a culture of ability, contrasting with more dominant deficit narratives. Mead theorised that, through this process, peer-support can lead to the collective construction of a new perspective on living with a condition. Similarly, Gillard et al. [62] identified the building of trusting relationships based on shared lived experience as a key mechanism in mental health peer-work. Whilst this was also a key element identified by Watson et al. [33], their review flagged that such mechanisms can contribute either positively or negatively to peer-support outcomes. Examples include instances where peers choose not to share their experiences leading to dissatisfaction from others about their lack of openness. Whilst no evidence of these negative outcomes was identified in our study, this may be related to the specific cohorts attending the observed courses. Formal evaluation at scale is needed to explore the potential for heterogeneity and negative outcomes in a more systematically selected sample.

The Good Life is a co-delivered model in which peer-tutors with lived experience are supported by trained facilitators. Price et al. [21] identified a dearth of evidence on professional facilitation of peer-support in health and social care. For dementia peer-support, this lack of evidence is particularly significant since it is likely that some degree of facilitation will always be necessary. Femiola and Tilki [27] noted that peer-led models have historically not been considered in dementia because of assumptions that cognitive impairment and memory problems would make peer-leadership unworkable. However, their research concluded that a peer-led approach was possible so long as peer-leaders were well supported. This concurs with our observation that, with person-centred facilitation, peer-tutors with dementia were able to effectively support others to learn about and discuss their dementia diagnosis. Moreover, Good Life peer-tutors reported finding the experience of being trusted to do this very rewarding. The degree of support required is likely to vary according to the confidence levels and capacities of individual peer-tutors, as well as the skills and experience of the facilitators and the degree of dementia progression [63]. Mason et al. [64] found that people with dementia participating in a professionally facilitated dementia peer-support group valued the facilitator’s role in maintaining ongoing communication, described as ‘keeping the pot boiling’. There is certainly a risk that programmes like the Good Life may be unsustainable if no attention is given to how professionals can support peer-leaders over time. Such professional involvement does not, in our interpretation, negate the innovative peer-led nature of the Good Life course, rather it is this support, tailored to the needs of the individual, which can make peer-leadership possible. Whilst a fully peer-led model (i.e., without any professional involvement) might be the aspiration for some groups, the Good Life with Dementia approach is generally one of partnership – doing *with.*

The value of providing support to enable people with dementia to take a lead themselves, in contrast to more traditional professionally led services, comes in part from the opportunities for meaningful roles and reciprocity this offers. Van Vliet, et al. [64] note the importance people with young-onset dementia place on staying engaged and feeling useful in the face of increasing impairment, and there is no reason to believe that older people with dementia would feel differently. Clare et al. [23] found that being part of a peer-support group helped people with young-onset dementia to feel like valuable members of society: ‘…*participants found that they had a purpose after diagnosis, were able to help others and acted as pioneers’* (p19), and Hagan and Campbell [24] noted similar for a group of people with dementia of mixed ages (up to age 80).

The presence of a different, less positive, view from one participant on the course demonstrates that a peer-led course will not be the preferred mode of support for everyone. This speaks to the ‘for whom’ question in realist evaluation: the same mechanism may not lead to the same outcomes for everyone. It is likely that a suite of options for post-diagnostic dementia support is required to meet the wide range of needs and preferences of the diverse range of people diagnosed with dementia: the Good Life course can only be one of these. The less satisfied Good Life participant pointed out that the course has primarily social benefits (which she referred to as ‘social shoring-up’) and cannot directly alter her dementia symptoms. Her disappointment at this highlights the importance of clear communication at the outset about the aims of the course and what it can and cannot offer. Social shoring-up could, nevertheless, be a valuable strategy in the recovery journey for some people as they seek to establish a new norm of life with a dementia diagnosis. In their review of everyday life after dementia diagnosis, Huizenga et al. [65] found multiple documented coping strategies including staying active, focusing on what can still be done, seeking social support and finding meaningful ways to (re)engage. In several of the studies they reviewed, a shift in attitude was reported towards appreciating the little things and living in the ‘now’. This supports the theoretical link made in our analysis between the social shoring-up offered by the Good Life course (i.e., the opportunity to build social connections and discuss experiences and feelings together, in a context of positive expectations) and the development of a new, more positive perspective on a future life with dementia.

### Strengths and limitations

A strength of this study is its novel combination of co-production and realist-informed methods. Whilst realist research has traditionally been researcher-led [44], there is a growing recognition that the inclusion of people with dementia on an equal footing has benefits both for people with dementia and for the quality of dementia research [46,47,66]. Our team took a pragmatic approach, with an emphasis on producing insights that would be useful to future researchers, practitioners and people living with dementia.

A limitation of our findings is that they are drawn primarily from the experiences of people who attended the two iterations of the course included in this study. This group was not representative of all people living with dementia, rather it consisted of people approached by virtue of their existing connections to services. We did not hear from people who were not invited to attend these Good Life courses (or indeed those who were invited but declined) and so we know very little about their views or reasons for declining. Despite a spread of gender, dementia type and age (61–81), there was no ethnic diversity within the interview sample, reflecting the lack of ethnic diversity in the self-selected population attending these courses (both iterations were attended exclusively by people identifying as White British). This is significant, since we know that stigma, culture and race can intersect to increase disadvantage for people with dementia from minoritised ethnic communities [67,68], and a lack of cultural inclusivity in health and social care provision can compound the general dearth of post-diagnostic dementia support [12]. This is something that it will be important to address in future research.

It should also be noted that we were unable to attend the first two sessions of GL1 due to delays in obtaining ethical approval, meaning we only observed five of the seven sessions in this first course. Whilst we do not think this will have substantially changed the findings, we cannot know what we missed in these first two sessions or whether this would have influenced our interpretation.

Finally, both courses we observed were facilitated by the same person, DM, who works for the community interest company that co-produced the original approach with people living with dementia. That facilitator was a co-applicant on the study and is a co-author on this paper, and it is important to recognise the conflicts and biases this potentially creates. The study was a collaborative endeavour bringing together impartial clinicians and academics with those with a stake in the model, having developed it or benefited directly from it (as previous participants or peer-tutors on the course). It would have been inappropriate, given our commitment to coproduction, for the academic team to divorce itself from the values and experiences of those with lived experience. Instead, we attempted to mitigate for the various positions on the team through our rigorous approach to testing initial assumptions and the wide range of stakeholders involved in theory refinement and consolidation. If future research seeks to establish a definitive answer on efficacy, however, the team roles would have to be more clearly delineated, with independent data collection and analysis.

### Implications and next steps

This study suggests that the Good Life with Dementia peer-led course could enhance personhood for people diagnosed with dementia, (re)building social confidence and connections, and helping participants to feel more positive about living with dementia. Distinguishing features of the approach include the core role of lived experience in the design and delivery of each iteration of the course, and the exclusive emphasis on people diagnosed with the condition being tutors and learners, not wider family or other carers. The core constructs and outcomes produced echo other research focusing on social health and the importance of this for people living with dementia [60].

To establish whether evidence of effectiveness can be collected in trial conditions, the next step will be a feasibility study for a randomised controlled trial of the Good Life approach against usual care. Building on findings of associated focus groups on the cultural transferability of the approach, this study will also explore the feasibility of trialling a Good Life course with people living with dementia from South Asian communities https://sscr.nihr.ac.uk/research/dementia/good-life-with-dementia/. Not everyone diagnosed with dementia will want to take part in a peer-led course, but interventions like the Good Life could be part of a suite of post-diagnostic support options available to help people with dementia to live as well as possible.

## Supporting information

S1 Fig Initial [pre-research] theory of change. (PDF)

S2 File Participant facing study information. (DOCX)

S3 File Capacity to consent form. (DOC)

S4 File Poster – This session is being observed. (PPTX)

S5 File I want to speak card. Available from Dementia Voices https://www.dementiavoices.org.uk/deep-resources/resources-for-deep-groups/admin-stuff/
(PDF)

S6 File Interview flashcards for Good Life research. (JPEG)

S7 File Topic guide for focus group. (DOCX)

S8 File Observation record – blank. (DOCX)
