# Supplementary figures and images for: The Good Life with Dementia approach: A realist-informed qualitative study of a peer-tutored course, co-produced with and for people living with dementia
Source: PLoS One. 2026 Jun 12;21(6):e0349444. doi: 10.1371/journal.pone.0349444 (PMC13262849; doi:10.1371/journal.pone.0349444)

# A GOOD LIFE WITH DEMENTIA - STAKEHOLDER-GENERATED THEORY OF CHANGE (PRE-RESEARCH)

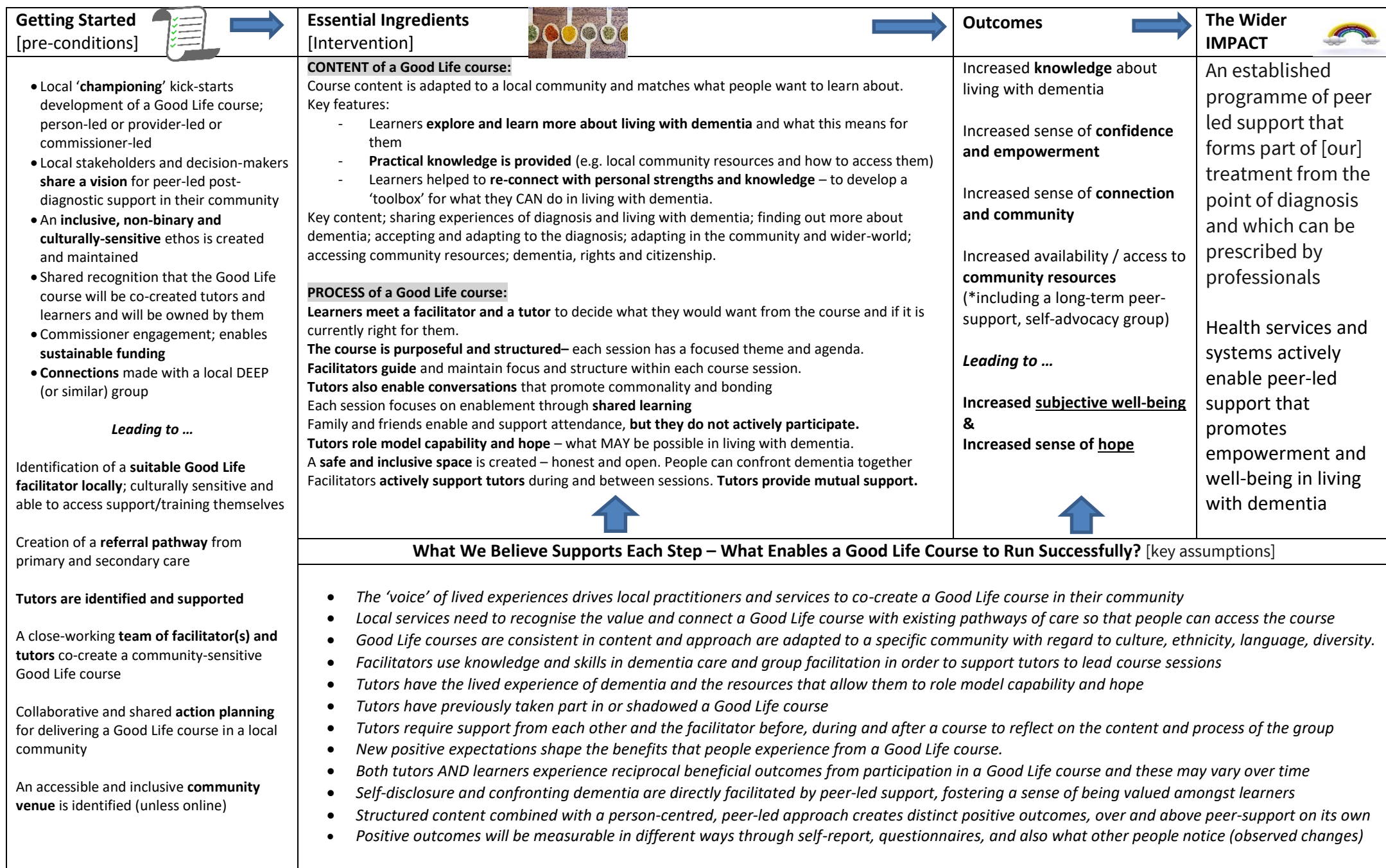

Supplement: S1 Fig — (PDF) [file pone.0349444.s001.pdf]

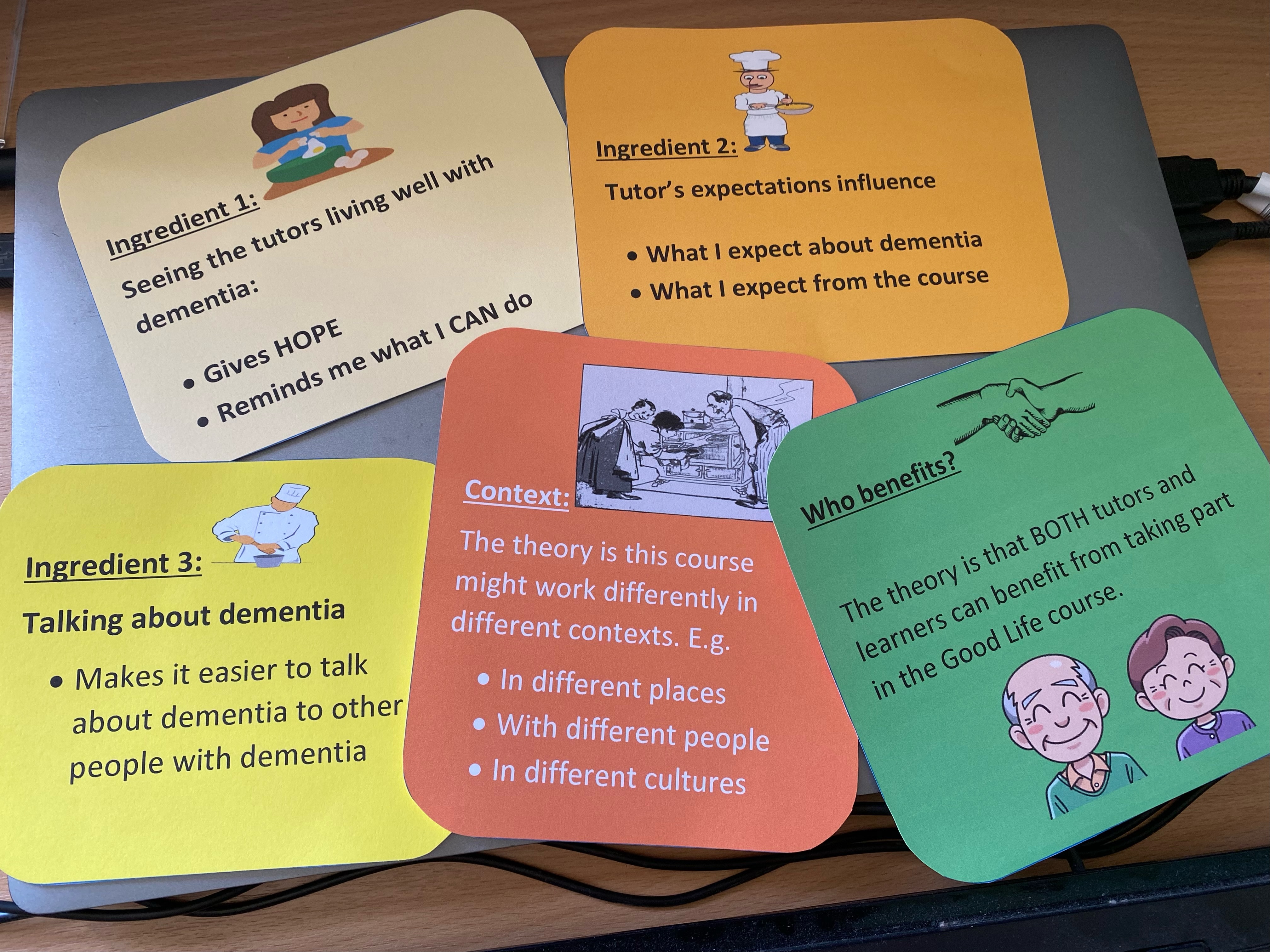

Supplement: S6 File — (JPEG) [file pone.0349444.s006.jpeg]
