# Supplementary material for: The Good Life with Dementia approach: A realist-informed qualitative study of a peer-tutored course, co-produced with and for people living with dementia
Source: PLoS One. 2026 Jun 12;21(6):e0349444. doi: 10.1371/journal.pone.0349444 (PMC13262849; doi:10.1371/journal.pone.0349444)
Supplement: S2 File — (DOCX) [file pone.0349444.s002.docx]

**Good Life Research Project**


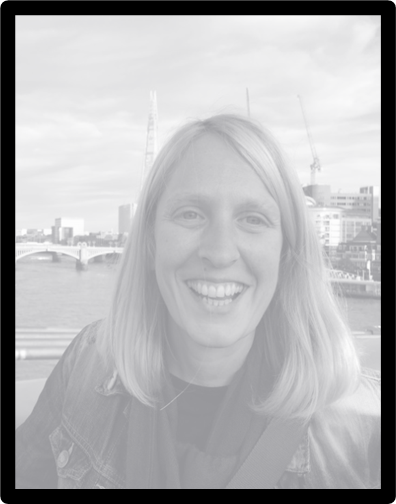
**Information Sheet**

**Replace with photo of researcher**

You are being invited to take part in some research about the Good Life with Dementia course looking at:

Hi, I’m [name]. I work as a researcher at the University of York. I’m working with the Good Life with Dementia team to understand how the course might help people and what could be improved.

I would like to observe and record some of the Good Life sessions so that I can learn from them. A co-researcher living with dementia will come too. Afterwards we will invite you to take part in a short interview and/or a group discussion with other people who have been on the Good Life course with you to find out about your views and experiences.

- What happens during the Good Life sessions?
- How do tutors and learners relate to each other?
- What is new or different about this type of course?
- How do you feel about the course?
- What could be done to improve the course for all?

This leaflet has more information about why we’re doing this research and how you could be involved. It’s up to you whether you take part in the research and to what extent. Some people might choose to take part in the interviews and be observed, others might not want us to use any information about them in the research, but might still be happy for us to observe and record sessions they are in. If you have any questions, please ask.

**What is the research about?** We want to learn more about the Good Life courses. How are these different from more traditional (professional-led) approaches to supporting people with dementia? What is it like for all the different people involved? How could the course be improved?

**Why have you been invited to take part?** You are either a Good Life course tutor or are thinking about attending a Good Life course.

With your permission, we would like to attend some of the Good Life sessions and observe and record what happens for our research. We are inviting you to take part in the research by allowing us to record and transcribe what you say when you attend the course. We would like to use this information to learn more about the Good Life approach. You can attend the Good Life course without taking part in the research. If you do sign up to the research, we may also invite you to take part in an interview and/or a group discussion. Again, you do not have to take part.

**What does taking part in the research involve?**

Observation for research purposes: We would like to come to some Good Life course sessions, take written notes and record (with a digital voice-recorder) what goes on. **For you to be included as a participant in the research we would need your consent to use your information to inform the evaluation**. All information will be treated in confidence.

Interview: After one of the Good Life sessions, we might invite you to take part in a short interview about your views and experiences of the course. There are no right or wrong answers - we want to hear what you think and feel in your own words. With your permission, we will voice-record these interviews so that we have a record of what is said. Interviews would take no longer than 30 minutes and you can take breaks whenever you want (or choose not to take part at all).

Group discussion: At the final Good Life session we will invite you to take part in a group discussion with other people who attended the course. We will ask everyone about their views and experiences of the Good Life course. With your permission, we will voice-record the discussion so that we have a record of what is said. This should take no longer than 50 minutes and you can take breaks whenever you want (or choose not to take part at all).

**What if I don’t want you to observe a session I’m in?** If you or anyone else in the session does not want us to observe and record then we won’t. The course facilitator will discuss this with you in advance, but you can also ask us to pause or stop recording during a session.

**What if I’m happy for you to observe and record a session, but don’t want to take part in the research myself?** You can agree to us observing and recording the sessions but still choose not to take part in the research yourself (see separate Agreement to be Observed and Recorded form). If you choose not to consent to take part in the research yourself, **we will not write down anything from or about you** and we won’t transcribe anything you say from our voice-recordings.

**Do you have to take part?** No. It is up to you to decide if you would like to take part in this research. If you decide you are happy to take part, you will be asked to complete a consent form (you can do this verbally if you’d prefer). You are free to change your mind about taking part at any time during the course of the research without giving a reason.

**Is everything confidential?** Yes. If you do decide to take part in this study, we will not tell anyone outside the research team without your permission, and you will not be identified in anything we write. If you tell us, during the course of our research, that you or someone else is being hurt, we might have to tell someone, but I would try to talk to you first.

The only person who will hear the voice-recordings (apart from the researchers) will be the person who transcribes them. This person has signed a data protection agreement.

**What happens to the information recorded?** We will save everything we record to the secure University of York system, which is password protected. This includes information about who you are, which will be kept there securely for up to a year after the study has finished. Information that does not have your name on it will be kept for 10 years.

At the end of the study some data may be kept anonymously so that other researchers can use it for future research. Your name will not be on any of this information. More information about data protection is available in the separate “data information sheet”.

**What will happen to the results of the study?** This research was co-produced with people living with dementia, and the study findings will also be co-produced. The results will be published in journals and presentations, and we will take advice from people living with dementia about how else we can publicise the findings to ensure the research reaches a wide range of different people that are involved in organising and delivering dementia care services.

**What are the possible benefits of taking part?** The research will not benefit you directly, but studying the Good Life course could help us to improve dementia support for people diagnosed with dementia in the future.

**Are there any risks to taking part?** You may find being observed makes you feel uncomfortable. If this happens just say and we can pause or stop recording.

You may be tired after a Good Life session and not want to take part in an interview. If you want to take a break or arrange to speak later that is fine, or you can decide not to do an interview at all.

**What if there is a problem?** If we have concerns about your wellbeing during the study, we will speak to you about this. Depending on the situation we may give you information about people who can help you, or contact them ourselves.

If at any time you decide you no longer want to take part please let us know. You can withdraw at any time. Any information we have already collected will still be used unless there is anything in particular you would like us to take out.

If you are not happy with any aspect of the study, please contact [name]

**Funding and ethical review:** This project was funded by the National Institute for Health Research: Three Schools Dementia Research. The study sponsor is the University of York [email] All documents and processes were reviewed and approved by the Health Research Authority (IRAS number 324935)

**For further information please contact name at the University of York**

**Email: [email] Tel: [number]**

**Thank you for reading this information sheet**

**Good Life Research Project**

**Information Sheet – Easy Read**

| **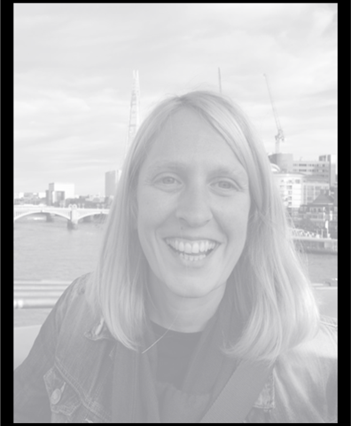[photo of researcher]** | Hello.  I am **[name]**  I am a researcher at the University of York.  My job is to learn about support for people with dementia.  I do this by listening to people with experience of dementia and by working together. |
| --- | --- |
| 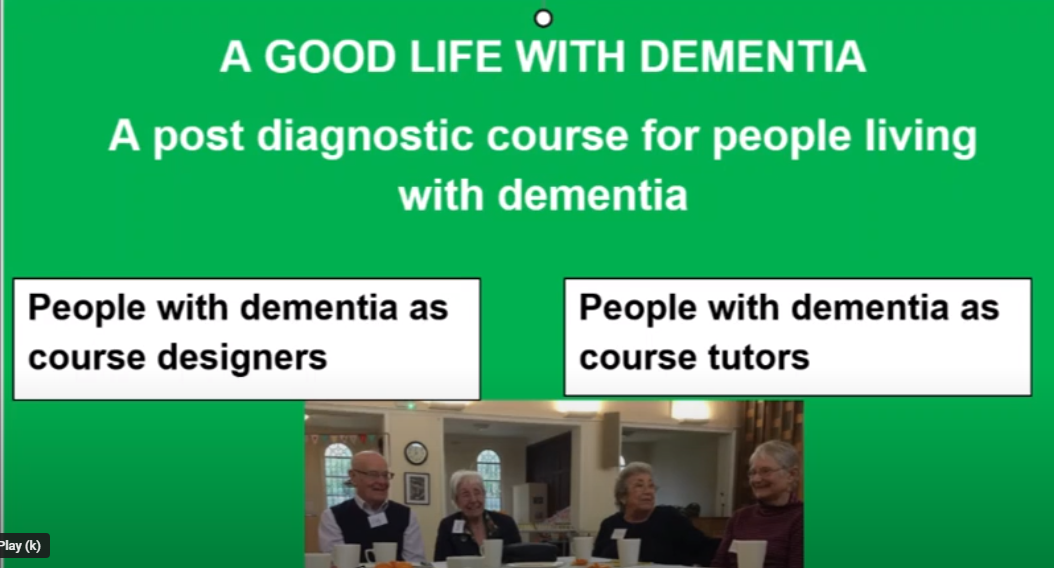 | **What is this study about?**  I am working with previous Good Life tutors to learn about the course.  Would you like to take part in our study? |
| 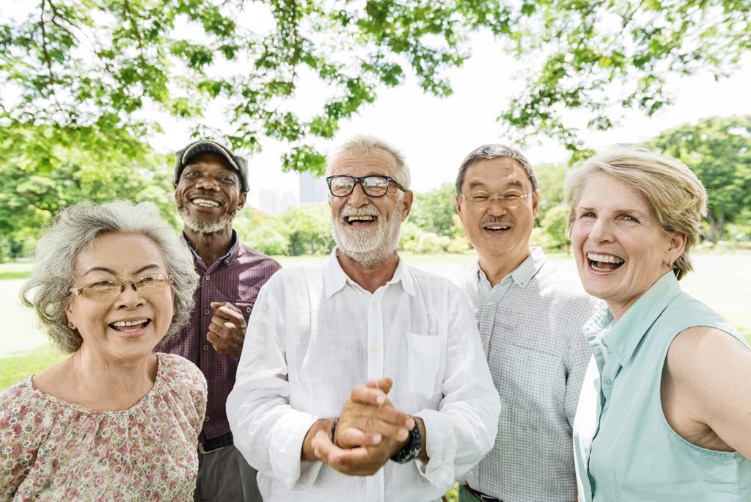 | **Who can take part in the study?**  People who are taking part in the Good Life course as tutors or learners. |
| 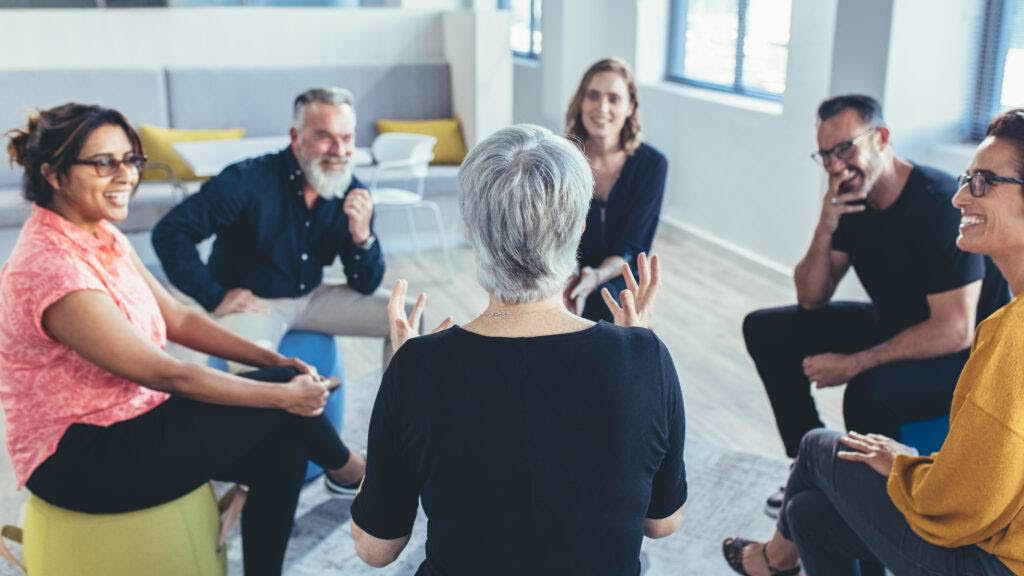 | **What will happen if I take part?**  We would like to observe and record some of the Good Life sessions.  If you consent to take part in the research, we might use information from you (anonymously) in the study.  You can still attend the course without being in the research. |
| 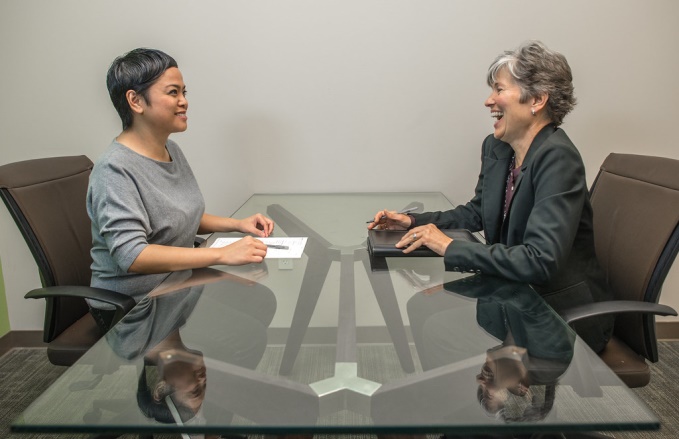 | We will invite you to take part in an interview and/or a group discussion with other people who attended the same Good Life course as you.  This will be voice-recorded with your permission. |
| 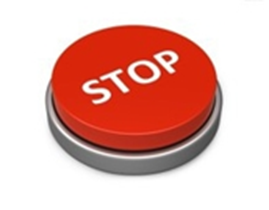 | **Do I have to take part?**  No.  It is up to you. |
| 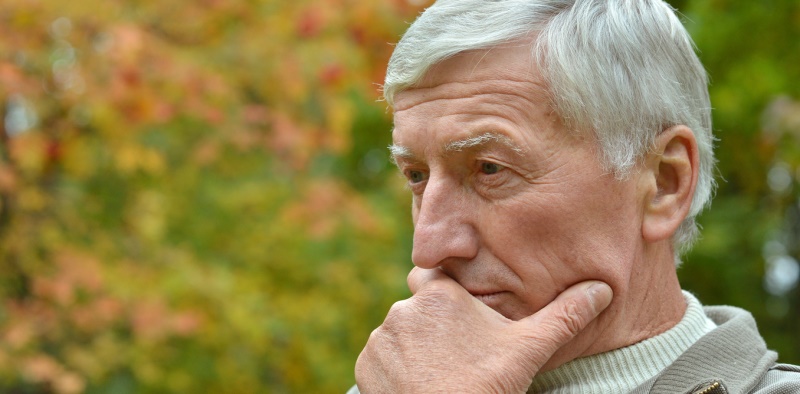 | **Can I change my mind?**  Yes. You can change your mind at any time by telling us. You don’t have to give a reason. |
| 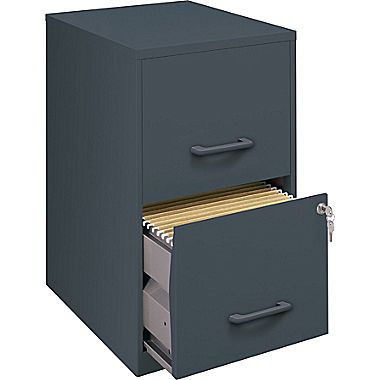 | **What happens to my information?**  Your information will be kept private and safe.  What we talk about will be recorded so we can remember it and make sure we get it right.  We won’t publish anything with your name on it. |
| 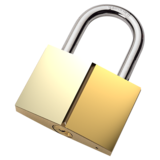 | Your personal information will only be seen by:   - People in the research team      - People who check the research is being done properly. |
| 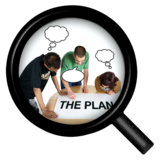 | **What will come from this research?**  We will work together to agree what we have learnt.  This will be written up and used to improve support for people with dementia. |
| 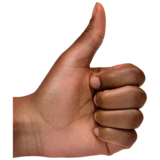  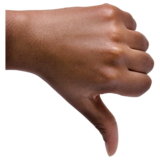 | **Good things about taking part?**   - You could be part of something that helps other people with dementia in the future   **Bad things about taking part?**   - You might not feel comfortable talking about some things in front of researchers   If you don’t like how something feels just say. We can stop or have a break. |
| Logos removed | **Who is organising this?**  The University of York are organising this study with the help of Innovations in Dementia.  An ethics committee has checked this study. |
| 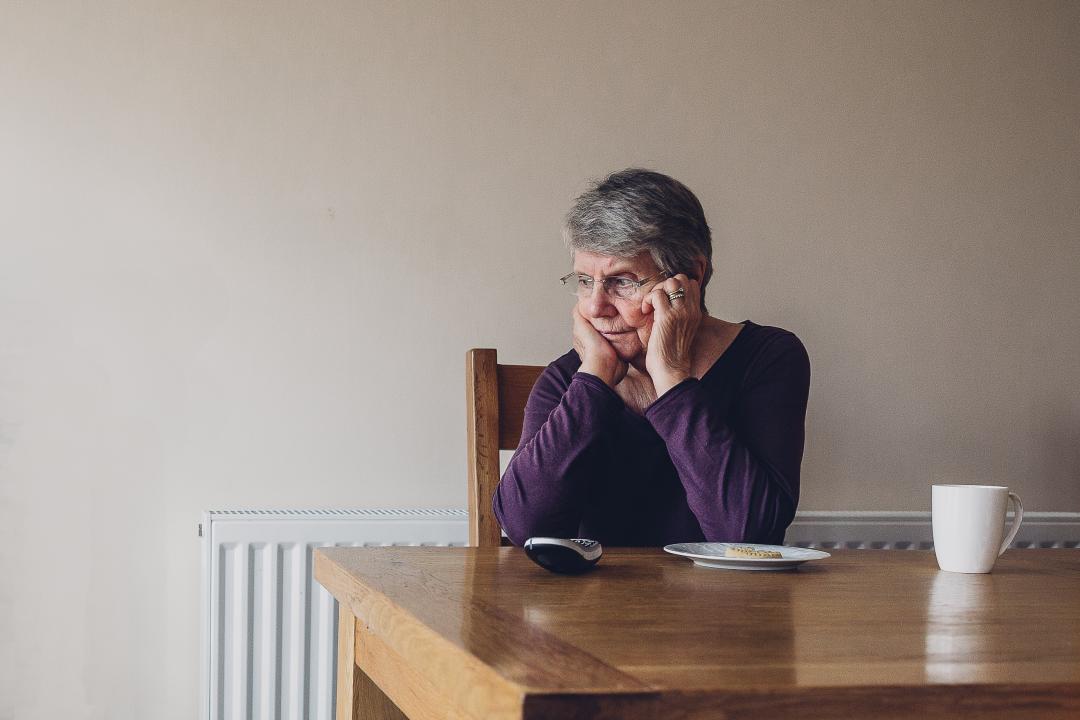 | **What if there is a problem?**  If you are not happy at any time please tell me.  If you want to complain formally you can do this too (see details below).  I may have to tell someone if I think you or someone else is not safe. I would talk to you about it first. |

| 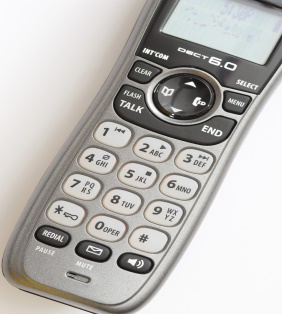  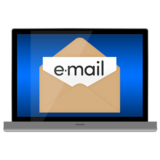  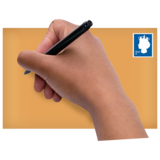 | **Researcher contact details**  Researcher: Name  **Telephone:** number  **Email:** [[name] @york.ac.uk](file:///F:\My%20Drive\1.%20WORK\A%20Good%20Life%20with%20Dementia\Ethics\Examples%20from%20other%20projects\lyndsay.lindley@york.ac.uk)  **To complain contact NAME** Tel: number  **Address:**  Address |
| --- | --- |
