# Supplementary material for: The Good Life with Dementia approach: A realist-informed qualitative study of a peer-tutored course, co-produced with and for people living with dementia
Source: PLoS One. 2026 Jun 12;21(6):e0349444. doi: 10.1371/journal.pone.0349444 (PMC13262849; doi:10.1371/journal.pone.0349444)
Supplement: S3 File — (DOC) [file pone.0349444.s003.doc]

**Participant identification number:** ⬜⬜⬜⬜⬜⬜⬜⬜⬜

**Assessment of Participant Capacity to Consent Form**

**Good Life in Dementia Research**

**(observation and interview)**

**TO BE COMPLETED BY RESEARCHER**

**Circle as appropriate**

**YES / NO**

1. Participant is able to understand the purpose of the study and what is involved

**YES / NO**

2. Participant is able to retain information long enough to make a decision

**YES / NO**

3. Participant is able to weigh up information in order to make a decision

4. Participant is able to communicate their decision

**YES / NO**

**It is my belief that the participant has the capacity to consent to take part in the study.**

**OR**

**It is my belief that the participant lacks the capacity to consent** (if answer is NO to any of Questions 1-4 above)

Name of participant: _______________________________

Name of Researcher: ­­­­­­­­­­­­­­­­­________________________________

Researcher Signature: ___________________ Date: ________________
