# Supplementary material for: The Good Life with Dementia approach: A realist-informed qualitative study of a peer-tutored course, co-produced with and for people living with dementia
Source: PLoS One. 2026 Jun 12;21(6):e0349444. doi: 10.1371/journal.pone.0349444 (PMC13262849; doi:10.1371/journal.pone.0349444)
Supplement: S4 File — (PPTX) [file pone.0349444.s004.pptx]

## Slide 1
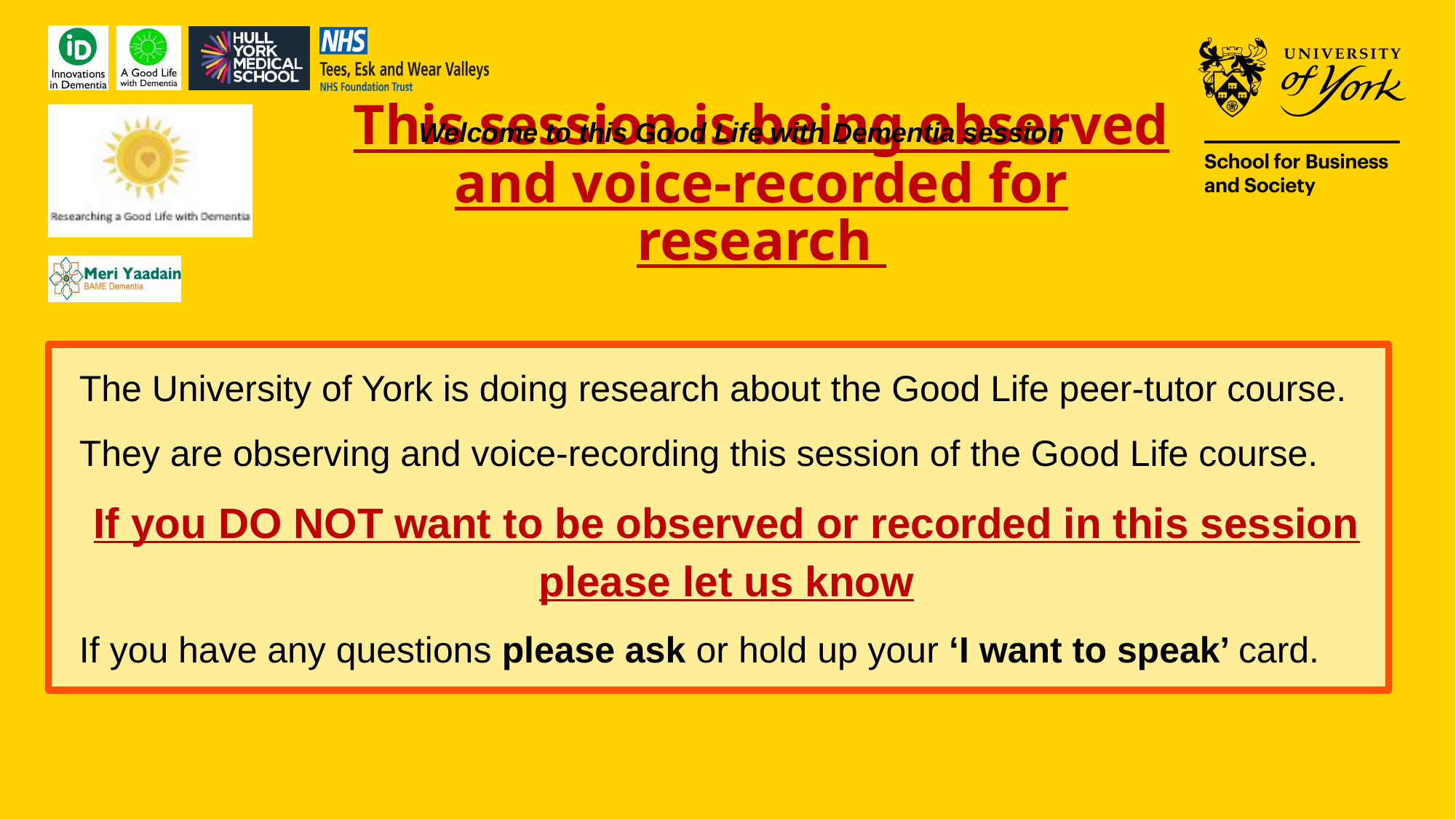

# This session is being observed and voice-recorded for research
Welcome to this Good Life with Dementia session
The University of York is doing research about the Good Life peer-tutor course.
They are observing and voice-recording this session of the Good Life course.
If you DO NOT want to be observed or recorded in this session please let us know
If you have any questions please ask or hold up your ‘I want to speak’ card.
