# Supplementary material for: The Good Life with Dementia approach: A realist-informed qualitative study of a peer-tutored course, co-produced with and for people living with dementia
Source: PLoS One. 2026 Jun 12;21(6):e0349444. doi: 10.1371/journal.pone.0349444 (PMC13262849; doi:10.1371/journal.pone.0349444)
Supplement: S5 File — Available from Dementia Voices https://www.dementiavoices.org.uk/deep-resources/resources-for-deep-groups/admin-stuff/ (PDF) [file pone.0349444.s005.pdf]

“I want to speak please”

“I want to speak please”
