# Supplementary material for: The Good Life with Dementia approach: A realist-informed qualitative study of a peer-tutored course, co-produced with and for people living with dementia
Source: PLoS One. 2026 Jun 12;21(6):e0349444. doi: 10.1371/journal.pone.0349444 (PMC13262849; doi:10.1371/journal.pone.0349444)
Supplement: S7 File — (DOCX) [file pone.0349444.s007.docx]

**Realist Focus Group Discussion about Good Life sessions**

**Aim:** The focus group should explore and test our candidate theories about why and how the peer-tutor approach works

Remember that for a realist focus group, we need to go through a cycle of:

1. Here is how we think the Good Life course achieves its intended effects.
2. Was that your experience? Why/ why not (or why 'only partially')
3. What have we missed? Are there important things going on here that we haven't asked you about?

**Topics about the key mechanisms (ingredients):**

**Topic 1. Tutors role modelling**

**We think that the GLC works because tutors on the course can show people who have recently been diagnosed with dementia how it’s possible to LIVE WELL with Dementia**

- **Does this affect/has this affected how any of you think about dementia?**
- **In what way? How?/ can you give me examples**

**We think this might help people feel more capable:**

- **In doing things**
- **In living with dementia**

**Has it made you feel more capable? In what ways/examples?**

**We think the course helps people feel that their life has hope/you can look forward to living life with dementia:**

- **Is this the case for you? Why? If not probe for reasons**
- **Are there any other ways it makes you feel? Probe for details**

**Are we missing anything? Is there anything else that the tutors do that anyone thinks is important? Examples?**

**Topic 2. Expectations**

**We think that the GLC works because it changes the expectations of people with dementia:**

- **Do you think your expectations have changed (of dementia/what dementia is/ how it affects you?)**
- **In what ways? Positive? Negative? Examples?**

**We think that these changes in expectations could help/encourage people to:**

1. **Keep taking part in/connect with the course/peer group**
2. **Feel better about the diagnosis**

- **Does anyone think that is the case? How? Examples?**

**Do you enjoy the weekly meetings/what is it you enjoy/ can you give me examples?**

- **Is there anything about the course you don’t enjoy? Probe for details and reasons**

**Are we missing anything?**

**Are there any other changes in other people’s expectations that anyone has noticed? E.g. family members/ friends**

**Topic 3. Self-disclosure**

**We think the GLC works:**

1. **Because it helps you to talk openly about your diagnosis/ dementia and what it means**
2. **Because it helps you confront/ come to terms with your diagnosis**

- **Do you think that is the case? How/can you give me examples**

**Topic 4: Shared experience**

**We think the GLC works:**

1. **Because the course is led by people with dementia, someone like yourselves, who has a similar or the same condition to you all have**
2. **Because you get to talk about shared experiences of what it is like to get a diagnosis / live with dementia**

- **Does anyone think that is the case? How/ can anyone give me examples?**
- **Are we missing anything?**

**Does anyone think there is anything about the course that makes it difficult:**

- **To talk openly about how they feel about your diagnosis**
- **To come to terms with your dementia diagnosis**

**BREAK WITH REFRESHMENTS**

**Topic 4. Context**

**Does anyone think that there might be some situations/circumstances where the GLC might not work for some people? Probe for details and reasons**

**What might make it work better?**

- **Is there anything that anyone can think of that could be done to improve the GLC for other people? Make it better?**
  - **E.g. Location/timings/different tutors/including people from different backgrounds/with different life experiences?**

**Topic 5. Outcomes**

**We think that both tutors and people taking part in the course could benefit from it:**

- **Does anyone think that this happens? How? In what way? Can anyone give examples?**
- **What sorts of benefits have you seen (for you/others)?**
- **Are there any drawbacks/negative effects of the course?**
- **What sorts of benefits would you like to see?**
- **How could we tell if this course ‘works’ for people with dementia (tutors and/or learners)?**

**Is there anything else that the GLC course helps with/ doesn’t help with that we haven’t talked about?**

**Thank you very much for your time today we really appreciate you all taking the time to take part in this group discussion and telling us all about your experiences of the GLC. It has been really helpful.**

**Thank you.**
