# Supplementary material for: The Good Life with Dementia approach: A realist-informed qualitative study of a peer-tutored course, co-produced with and for people living with dementia
Source: PLoS One. 2026 Jun 12;21(6):e0349444. doi: 10.1371/journal.pone.0349444 (PMC13262849; doi:10.1371/journal.pone.0349444)
Supplement: S8 File — (DOCX) [file pone.0349444.s008.docx]

**GOOD LIFE Observation notes**

**Researcher participant ID: ________________ GLC session ID: _____________________**

**ID other: _____________________Date and stage of GL data collection: _______________**

| **Behaviour (individual)** |  |
| --- | --- |
| **Interactions**  **(Individuals relating to each other)** |  |
| **Body Language** |  |
| **Other** |  |

| **Time** | **Role modelling** | **Expectations** | **Self-disclosure** | **Context** | **Outcomes** | **Relating** | **Other observations** |
| --- | --- | --- | --- | --- | --- | --- | --- |
|  |  |  |  |  |  |  |  |

**Overall** (Note here any overall impressions/observations not noted elsewhere):
