# Supplementary material for: The Good Life with Dementia approach: A realist-informed qualitative study of a peer-tutored course, co-produced with and for people living with dementia
Source: PLoS One. 2026 Jun 12;21(6):e0349444. doi: 10.1371/journal.pone.0349444 (PMC13262849; doi:10.1371/journal.pone.0349444)
Supplement: Attachment — Submitted filename: Response to Reviewers 27th March 2026.docx [file pone.0349444.s010.docx]

27^th^ March 2026

Kate Gridley

University of York

[kate.gridley@york.ac.uk](mailto:kate.gridley@york.ac.uk)

Dear members of the PLOS One Editorial Board and Reviewers,

Thank you for taking the time to review this submission. We are encouraged by your feedback and have found your suggestions very useful.

Please find below a point-by-point outline of how we have responded to your comments. If you require any further information please do not hesitate to come back to us.

Yours faithfully,

Kate Gridley (on behalf of the Good Life with Dementia Research Team)

[Kate.gridley@york.ac.uk](mailto:Kate.gridley@york.ac.uk)

# Editor’s letter

| **Editor’s instructions** | **Response** |
| --- | --- |
| Please take careful note of the author's requests for methodological clarity. This is a timely and important contribution to the literature, and so clear details in this section will improve the quality of the submission. | Thank you for these encouraging comments. We have added detail to the background and methods sections as requested by the reviewers, covering:   - Consent process - Capacity to consent and stage of dementia - Numbers of participants involved in different elements of the fieldwork - Data collection methods and the rationale behind these - Our inclusive approach - Ensuring rigour in analysis |
| Regarding the reviewers' comments to highlight literature in other populations, I would encourage   - including a brief overview of chronic health challenge peer support - and mental health peer support and the recovery movement. We have added a section on dementia recovery colleges   The goals in this kind of peer support are often very different from the kinds of goals your participants articulated, but the idea of living well with mental or chronic physical illness would be relevant. | This is a helpful suggestion. We have added a section to the ‘Background and introduction’ on the theory underpinning peer-support in other populations, which includes a paragraph on the recovery movement and dementia specific Recovery College courses (see ‘Peer-support: Theory and Evidence’). We have also returned to this in the Discussion, noting similarities between our refined programme theory and the principles underpinning recovery. |

## Review Comments to the Author

### Reviewer #1:

This is a very interesting study that makes important contributions to the evidence base for post-diagnostic and peer support for people living with dementia. Below are suggestions to help strengthen the manuscript.

| **Background and Introduction:** |  |
| --- | --- |
| This section could be expanded to better situate the study within existing literature and clearly articulate the evidence gap. In particular, further discussion of the extensive peer-support literature in other populations, contrasted with the more limited evidence for people living with dementia, would help readers understand the significance of the study. | Thank you for this suggestion – we have added an additional section to the Background and introduction called ‘Peer-support: Theory and Evidence’ (page 4, line 82) |
| Additional detail about the Good Life with Dementia course itself, especially its core structure and components, would also be helpful | We have added more detail about the Good Life course itself to the section of the Background and introduction with the title: ‘The Good Life with Dementia course’ |
| **Methodology and Methods:** |  |
| Recruitment and consent: More clarity is needed regarding the consent process. Please describe how capacity to provide informed consent was assessed and whether information on participants’ stage of dementia was collected. If individuals living with moderate to severe dementia were excluded, provide a clear rationale. Consider including examples of visual prompts used during recruitment, either in the text or as supplementary material. | We have added a more detailed description of the consent process including how mental capacity was assessed (see tracked changes and references to supplementary information in ‘Recruitment and consent’ page 9, lines 205 to 219). Information on stage of dementia was not collected as the pertinent information was felt to be capacity to understand and engage, which is not always related to clinical stage of dementia. |
| Data collection: The manuscript notes that researchers observed all but the first two sessions due to delayed ethical approval. Please discuss how this may have influenced the findings, potentially in the limitations section. Clarify the total number of interviews and focus groups conducted and whether all participants took part in the focus groups. | We observed 5/7 sessions of the first course (GL1), and the full 6 sessions of the second course (GL2). We do not think that missing the first two sessions of GL1 will have substantially influenced the findings. Five weeks of observation gave sufficient time for the researchers to observe change in group dynamic and individual confidence, and all participants were still invited to be interviewed towards the end of the course. However, we have made a note in the Limitations section of the manuscript (page 33, line 750 – 754) acknowledging that we cannot know what we missed in these first two sessions or whether this would have influenced our interpretation.  We have added numbers to the ‘Data collection’ section in Methods (page 10, lines 230-245), making it clear that on GL1 there were 14 participants (12 learners and 2 tutors) 8 of whom were interviewed, and on GL2 there were 8 participants (7 learners and 1 tutor) 6 of whom were interviewed. Whilst not all participants were interviewed, all gave informed consent and all took part in an end of course discussion. |
| The description of interviews would benefit from further elaboration on the realist interviewing techniques and principles of inclusive dementia research used. When noting that follow-up interviews were conducted “soon after,” please specify the timeframe (e.g., within 1–2 days). | Further information has been added about the approach to interviewing, as well as an example question and link to visual aids in supplementary information (page 11, lines 261-271). We have clarified that whilst most interviews were undertaken during or immediately after sessions, two interviews were conducted nearly 2 months later (on the request of the participants). |
| Data Analysis: Please describe how rigor was ensured during the study. | To ensure rigour we followed the four fundamentals of realist analysis (1) simultaneous data collection and analysis, (2) retroductive theorising, (3) configurational analysis and (4) realist analysis quality (Rees, C. E., Proctor, D. W., Nguyen, V. N., Ottrey, E., & Mattick, K. L. (2025). Realist analysis of qualitative data in health professions education research. Medical Education, 59(5), 503-518.) We have added text to the start of the Analysis section (page 12, lines 282-304) setting out how elements of our approach map to these fundamentals and continuing to explain that the latter can be broken down into trustworthiness, coherence and plausibility. Trustworthiness was promoted through our iterative approach to theory building and testing, and the meaningful involvement of people with lived experience in all stages of this. Coherence and plausibility were further promoted in the theory refinement and consolidation stages where candidate elements of the theory were presented to those with expertise for comment and duly developed. Indeed, a key component of our strategy to ensure rigour comes from our inclusion of a range of voices and types of expertise, including professional and lived experience, in all stages of the process. Co-researchers and the wider advisory group were involved in discussions to refine the theory at key stages in analysis, and towards the end of the study we held a two-day residential to bring these voices together and consolidate our interpretation. This strategy is already described in the paper. However, we have added more detail in the Analysis section (page 12, lines 284-286) about the collaborative construction of the framework (which involved four researchers regularly meeting to discuss interpretation) as this was another measure which ensured that no single voice or perspective dominated the analysis. |
| Discussion Consider integrating literature on similar peer-led courses or programs to further support and strengthen the conclusions. | We have added a note about the similarities and differences between Good Life and Recovery College dementia courses to the Discussion (page 29, lines 653-657) |
| Additionally, the statement on lines 595–596 (“In several of the studies reviewed…”) requires an appropriate citation to substantiate the claim. | This statement relates to the Huizenga et al. paper cited earlier in the paragraph – we have made this clearer in the manuscript |

## Reviewer #2:

Thank you for the opportunity to read this paper. Post-diagnostic support for people living with dementia is an important and timely issue. I would recommend addressing the following issues before publication in PLOS One:

| Although the theory of change is integrated throughout the manuscript, there should be more description to ensure readers have sufficient information to understand your approach. | We have described the course in more detail (pages 5-7, lines 117-155) and made it clear (page 7, line 162) that in this research we were building on an initial (pre-research) theory of change generated by stakeholders (S1). The refined programme theory that resulted from the research is described throughout the findings and set out pictorially in Figure 2. It sounds like this did not upload successfully in our initial submission – apologies for this, we have now included it as an image at the end of the manuscript and will endeavour to ensure upload of the Figure2 file is successful. |
| --- | --- |
| **Methodology and methods:** |  |
| There is a lack of information about the study design. Interviews and focus groups were part of the methods used in this study; however, there is limited information on the kinds of interviews and focus groups that were conducted. At one point, the interviews were described as "realist-informed interviews," but there should be more information to explain what that means in your research. The manuscript would benefit from more information to distinguish the purpose of the interviews compared to the focus groups and what kinds of interview questions and focus group prompts were included (and refined between GL1 and GL2). | As noted above, we have added more detail to the Data collection section illustrating our realist-informed, inclusive approach to the interviews (page 11, lines 254-275)  The group discussion at the end of GL1 was led by the course facilitator (a co-applicant on the research) working to a list of questions provided by the research team. The data from this was so useful that we submitted an ethics amendment between GL1 and GL2 allowing a researcher to run a formal focus group at the end of GL2. The topic guide for this covered similar topics to the interview topic guide, but instead of visual aids the scaffolding came from the supportive group environment. We have included the focus group topic guide in Supplementary information (S7. Topic guide for focus group) |
| Any software/coding usage, participant checking, duration and setting of the interview would be some useful information. | A note has been added to the Methods (page11, lines 264-266) to say that interviews were held in the community setting where the courses ran and ranged from short (micro) interviews in session breaks to full length interviews after the sessions (in accordance with participants’ preferences).  The only software used for analysis was Excel for Framework, stored on a shared (secure) drive so that multiple researchers could contribute to the same documents.  Re participant checking, people with lived experience, including study participants, attended an in-person knowledge exchange event towards the end of the study (after the analysis residential) where they were invited to comment on findings for final consolidation. |
| Additionally, there is a lack of information about the observations and the creation of field notes. | More detail about the recording of observations and a reference to the observation template used by academic researchers (S8. Observation record – blank) has been added to the Data Collection section (pages 10-11, lines 251-53). |
| Also, one article was cited to support the use of the principles of inclusive dementia research, but this does not provide adequate information on which/how these principles were used in this study. Visual aids were also mentioned (see lines 177-178), but the purpose of these was not clearly explained, such as what these visual aids entailed. | Further information has been added (page 11, lines 259-267) about our approach to inclusive dementia research, which is grounded in the idea of ‘fit’ and ‘misfit’ and requires flexibility in the field, as well as meaningful involvement in study design. An additional reference has been added (Webb, J., Williams, V., Gall, M., & Dowling, S. (2020). Misfitting the research process: Shaping qualitative research “in the field” to fit people living with dementia. International Journal of Qualitative Methods, 19, 1609406919895926.) and we have uploaded an image showing the visual aids used as supplementary information (S6. Interview flashcards for Good Life research). |
| Citations should be included to support the sentences about a realist ontology (see lines 118-119) and the epistemological approach that was used (see lines 124-126). | The reference for this is *Jagosh J. Realist synthesis for public health: building an ontologically deep understanding of how programs work, for whom, and in which contexts. Annu Rev Public Health. 2019;40:361-72.* We have moved this citation to the start of this paragraph (page 7, line 177) and have repeated it at the end to show clearly that the full paragraph draws on this text. |
| Supplementary material: The manuscript refers to “Supplementary Document A” (see lines 105 and 233), and two figures were also captioned in the manuscript. It appears that not all of this material was provided. Only one supplementary document titled “S1. Fig initial (pre-research) theory of change” was provided. This S1 document was well designed. | It is not clear to us why the two figures did not upload – these visual representations are important aids to understanding the programme theory. We will endeavour to upload them successfully on resubmission and we have also included them as pictures at the end of the manuscript so that we can guarantee that they are visible to reviewers. |
| The analysis of the refined programme theory is strong, particularly for the reflection on the concept of hope. This manuscript also showcased transparency by including the 'discordant views' that came up. My recommendation for this section is to clearly explain 'social shoring-up' as it came up two more times. | Thank you for these encouraging comments.  The term ‘social shoring up’ came from the participant with discordant views, who was contrasting this social focus with more medical interventions that might, in theory, influence the clinical symptoms of dementia. To us this distinction is not unhelpful since the course does indeed focus on what can be influenced socially, which could in turn have benefit for people’s future experience of living with dementia. We have added a line to the Discussion (page 32, lines 725-6) making it clear how we are interpreting the term ‘social shoring up’ (i.e. the opportunity to build social connections and discuss experiences and feelings together, in a context of positive expectations). |
| The analysis also includes a summary of the consolidated programme theory, which includes themes on shared experience, equality, and positive expectations. However, equality was not well established in relation to the data, and at one point seemed to be conflated with “sameness” (see line 463). I would recommend strengthening the analysis of equality to offer a strong conceptual advance and a robust evaluation in the context of dementia. | On reflection we can see that the use of the term ‘sameness’ could be misleading. Some participants did specifically refer to being ‘all the same’ but clearly there were many differences between participants too. Our interpretation is that, when peers referred to ‘being in the same boat’ they were referring to the camaraderie that comes from shared experience. To the section in the Discussion that deals with equality (pages 28 and 29, lines 629-646) we have added two references which present evidence for the role of camaraderie and ‘common humanity’ in peer-support, in contrast to previous theorising that difference (and in particular social comparison) was the key mechanism for positive outcomes (something our evidence does not support). |
| Additional note: Under observed outcomes (on line 248), there is an extra period before the sentence "Figure 1" starts. | Thank you for pointing this out, it has now been deleted. |

Journal Requirements:
When submitting your revision, we need you to address these additional requirements.

| Requirements | Response |
| --- | --- |
| 1. Please ensure that your manuscript meets PLOS ONE's style requirements, including those for file naming. (see email for links to templates) | We believe that it does |
| 2. Please provide a complete Data Availability Statement in the submission form, ensuring you include all necessary access information or a reason for why you are unable to make your data freely accessible. | We would like to seek an exemption on the grounds that it is not possible to ensure the anonymity of participants given that this research is based on data collected at two unique iterations of a course that ran on specific dates (when no other Good Life courses were running in the country). |
| 3. When completing the data availability statement of the submission form, you indicated that you will make your data available on acceptance. We strongly recommend all authors decide on a data sharing plan before acceptance, as the process can be lengthy and hold up publication timelines. Please note that, though access restrictions are acceptable now, your entire data will need to be made freely accessible if your manuscript is accepted for publication. This policy applies to all data **except where public deposition would breach compliance with the protocol approved by your research ethics board.** If you are unable to adhere to our open data policy, please kindly revise your statement to explain your reasoning and we will seek the editor's input on an exemption. Please be assured that, once you have provided your new statement, the assessment of your exemption will not hold up the peer review process. | We had initially planned to upload anonymised transcripts to an open repository but, after discussion with colleagues, we do not think these data should be made available because we cannot guarantee anonymity even after transcripts have been de-identified (because of the small pool of people who have ever been on this course) and as such, public deposition would breach compliance with our ethical approvals. |
| 4. We note that you have provided funding information that is not currently declared in your Funding Statement. However, funding information should not appear in the Acknowledgments section or other areas of your manuscript. We will only publish funding information present in the Funding Statement section of the online submission form. | We have removed the funding information from the Acknowledgements |
| Please remove any funding-related text from the manuscript and let us know how you would like to update your Funding Statement. Currently, your Funding Statement reads as follows: This independent research was funded by the National Institute for Health and Care Research (NIHR) on behalf of the NIHR Three Schools' Dementia Research Programme. KG was the Lead Researcher (for details of the full team see <https://sscr.nihr.ac.uk/research/dementia/good-life-with-dementia/>). The views expressed are those of the authors and not necessarily those of the NIHR or the Department of Health and Social Care. The funder played no role in the study design, data collection and analysis, decision to publish, or preparation of the manuscript. | We have removed funding-related text from the manuscript/acknowledgements. The text of the funding statement should read: ‘This independent research was funded by the National Institute for Health and Care Research (NIHR) on behalf of the NIHR Three Schools' Dementia Research Programme. The views expressed are those of the authors and not necessarily those of the NIHR or the Department of Health and Social Care. KG was the Lead Researcher (for details of the full team see <https://sscr.nihr.ac.uk/research/dementia/good-life-with-dementia/>). The funder played no role in the study design, data collection and analysis, decision to publish, or preparation of the manuscript.’ |
| 5. Please amend your list of authors on the manuscript to ensure that each author is linked to an affiliation. Authors’ affiliations should reflect the institution where the work was done (if authors moved subsequently, you can also list the new affiliation stating “current affiliation:….” as necessary). | We emailed you to check this and you confirmed that our affiliations do not need to be modified |
| 6. Please upload a copy of Figure 1 and 2, to which you refer in your text on page 13 and 23. If the figure is no longer to be included as part of the submission please remove all reference to it within the text | These were uploaded but we have removed them and will attempt to upload them again. **Please do contact us if you do not receive them** as they are essential to comprehension on the article. |
| 7. We note that Figure(s) S1 in your submission contain copyrighted images. All PLOS content is published under the Creative Commons Attribution License (CC BY 4.0), which means that the manuscript, images, and Supporting Information files will be freely available online, and any third party is permitted to access, download, copy, distribute, and use these materials in any way, even commercially, with proper attribution. For more information, see our copyright guidelines: <http://journals.plos.org/plosone/s/licenses-and-copyright>. We require you to either (1) present written permission from the copyright holder to publish these figures specifically under the CC BY 4.0 license, or (2) remove the figures from your submission: | S1 is the original (pre-research) theory of change developed by stakeholders with facilitation from the team that went on to undertake the Good Life research. As such it was not a copyrighted image (and we do have the permission of the team to use it). |
| 8. Please include captions for your Supporting Information files at the end of your manuscript, and update any in-text citations to match accordingly. Please see our Supporting Information guidelines for more information: <http://journals.plos.org/plosone/s/supporting-information>. | These are now at the end of the manuscript |
| 9. If the reviewer comments include a recommendation to cite specific previously published works, please review and evaluate these publications to determine whether they are relevant and should be cited. There is no requirement to cite these works unless the editor has indicated otherwise. | N/A |
| 10.Please review your reference list to ensure that it is complete and correct. If you have cited papers that have been retracted, please include the rationale for doing so in the manuscript text, or remove these references and replace them with relevant current references. Any changes to the reference list should be mentioned in the rebuttal letter that accompanies your revised manuscript. If you need to cite a retracted article, indicate the article’s retracted status in the References list and also include a citation and full reference for the retraction notice. | There have been substantial changes to the reference list, with several new citation added in response to reviewers’ comments, and these are all marked as tracked changes in the tracked version of the manuscript. |
