# Supplementary material for: The Good Life with Dementia approach: A realist-informed qualitative study of a peer-tutored course, co-produced with and for people living with dementia
Source: PLoS One. 2026 Jun 12;21(6):e0349444. doi: 10.1371/journal.pone.0349444 (PMC13262849; doi:10.1371/journal.pone.0349444)
Supplement: Peer review history

# Peer review: Decision Letter 0

**DOI:** 10.1371/journal.pone.0349444.r001

**Authors:** Patricia Anne Morris

19 Feb 2026

PONE-D-25-57625

The Good Life with Dementia Approach: A realist-informed qualitative study of a peer-tutored course, co-produced with and for people living with dementia.

PLOS One

Dear Dr. Gridley,

Thank you for submitting your manuscript to PLOS ONE. After careful consideration, we feel that it has merit but does not fully meet PLOS ONE’s publication criteria as it currently stands. Therefore, we invite you to submit a revised version of the manuscript that addresses the points raised during the review process.

==============================

Please take careful note of the author’s requests for methodological clarity. This is a timely and important contribution to the literature, and so clear details in this section will improve the quality of the submission. Regarding the reviewers’ comments to highlight literature in other populations, I would encourage including a brief overview of chronic health challenge peer support and mental health peer support and the recovery movement. The goals in this kind of peer support are often very different from the kinds of goals your participants articulated, but the idea of living well with mental or chronic physical illness would be relevant.

==============================

Please submit your revised manuscript by Apr 05 2026 11:59PM. If you will need more time than this to complete your revisions, please reply to this message or contact the journal office at plosone@plos.org. When you’re ready to submit your revision, log on to https://www.editorialmanager.com/pone/ and select the ‘Submissions Needing Revision’ folder to locate your manuscript file.

Please include the following items when submitting your revised manuscript:

- A letter that responds to each point raised by the academic editor and reviewer(s). You should upload this letter as a separate file labeled ‘Response to Reviewers’.
- A marked-up copy of your manuscript that highlights changes made to the original version. You should upload this as a separate file labeled ‘Revised Manuscript with Track Changes’.
- An unmarked version of your revised paper without tracked changes. You should upload this as a separate file labeled ‘Manuscript’.

-->If you would like to make changes to your financial disclosure, please include your updated statement in your cover letter. Guidelines for resubmitting your figure files are available below the reviewer comments at the end of this letter.

If applicable, we recommend that you deposit your laboratory protocols in protocols.io to enhance the reproducibility of your results. Protocols.io assigns your protocol its own identifier (DOI) so that it can be cited independently in the future. For instructions see: https://journals.plos.org/plosone/s/submission-guidelines#loc-laboratory-protocols. Additionally, PLOS ONE offers an option for publishing peer-reviewed Lab Protocol articles, which describe protocols hosted on protocols.io. Read more information on sharing protocols at https://plos.org/protocols?utm_medium=editorial-email&utm_source=authorletters&utm_campaign=protocols.

We look forward to receiving your revised manuscript.

Kind regards,

Patricia Anne Morris

Academic Editor

PLOS One

Journal Requirements:

When submitting your revision, we need you to address these additional requirements.

1. Please ensure that your manuscript meets PLOS ONE’s style requirements, including those for file naming. The PLOS ONE style templates can be found at

https://journals.plos.org/plosone/s/file?id=wjVg/PLOSOne_formatting_sample_main_body.pdf and https://journals.plos.org/plosone/s/file?id=ba62/PLOSOne_formatting_sample_title_authors_affiliations.pdf

2. Please provide a complete Data Availability Statement in the submission form, ensuring you include all necessary access information or a reason for why you are unable to make your data freely accessible. If your research concerns only data provided within your submission, please write "All data are in the manuscript and/or supporting information files" as your Data Availability Statement.

3. When completing the data availability statement of the submission form, you indicated that you will make your data available on acceptance. We strongly recommend all authors decide on a data sharing plan before acceptance, as the process can be lengthy and hold up publication timelines. Please note that, though access restrictions are acceptable now, your entire data will need to be made freely accessible if your manuscript is accepted for publication. This policy applies to all data except where public deposition would breach compliance with the protocol approved by your research ethics board. If you are unable to adhere to our open data policy, please kindly revise your statement to explain your reasoning and we will seek the editor’s input on an exemption. Please be assured that, once you have provided your new statement, the assessment of your exemption will not hold up the peer review process.

4. Thank you for stating the following in the Acknowledgments Section of your manuscript:

This independent research was funded by the National Institute for Health and Care Research (NIHR) on behalf of the NIHR Three Schools’ Dementia Research Programme. The views expressed are those of the authors and not necessarily those of the NIHR or the Department of Health and Social Care. We acknowledge the valuable contributions of all those involved, in particular people living with dementia who took part in the research. This paper is dedicated to the memory of Wendy Mitchell, who was an expert by experience and co-researcher on the project, and played a key role in developing the Good Life with Dementia approach from the beginning. Wendy’s contribution to research was one of many ways in which she made a positive impact and inspired others. Her wisdom and insights permeate all elements of this research.

We note that you have provided funding information that is not currently declared in your Funding Statement. However, funding information should not appear in the Acknowledgments section or other areas of your manuscript. We will only publish funding information present in the Funding Statement section of the online submission form.

Please remove any funding-related text from the manuscript and let us know how you would like to update your Funding Statement. Currently, your Funding Statement reads as follows:

This independent research was funded by the National Institute for Health and Care Research (NIHR) on behalf of the NIHR Three Schools’ Dementia Research Programme. KG was the Lead Researcher (for details of the full team see https://sscr.nihr.ac.uk/research/dementia/good-life-with-dementia/). The views expressed are those of the authors and not necessarily those of the NIHR or the Department of Health and Social Care. The funder played no role in the study design, data collection and analysis, decision to publish, or preparation of the manuscript.

Please include your amended statements within your cover letter; we will change the online submission form on your behalf.

5. Please amend your list of authors on the manuscript to ensure that each author is linked to an affiliation. Authors’ affiliations should reflect the institution where the work was done (if authors moved subsequently, you can also list the new affiliation stating “current affiliation:….” as necessary).

6. Please upload a copy of Figure 1 and 2, to which you refer in your text on page 13 and 23. If the figure is no longer to be included as part of the submission please remove all reference to it within the text.

7. We note that Figure(s) S1 in your submission contain copyrighted images. All PLOS content is published under the Creative Commons Attribution License (CC BY 4.0), which means that the manuscript, images, and Supporting Information files will be freely available online, and any third party is permitted to access, download, copy, distribute, and use these materials in any way, even commercially, with proper attribution. For more information, see our copyright guidelines: http://journals.plos.org/plosone/s/licenses-and-copyright.

We require you to either (1) present written permission from the copyright holder to publish these figures specifically under the CC BY 4.0 license, or (2) remove the figures from your submission:

a. You may seek permission from the original copyright holder of Figure(s) S1 to publish the content specifically under the CC BY 4.0 license.

We recommend that you contact the original copyright holder with the Content Permission Form (http://journals.plos.org/plosone/s/file?id=7c09/content-permission-form.pdf) and the following text:

“I request permission for the open-access journal PLOS ONE to publish XXX under the Creative Commons Attribution License (CCAL) CC BY 4.0 (http://creativecommons.org/licenses/by/4.0/). Please be aware that this license allows unrestricted use and distribution, even commercially, by third parties. Please reply and provide explicit written permission to publish XXX under a CC BY license and complete the attached form.”

Please upload the completed Content Permission Form or other proof of granted permissions as an "Other" file with your submission.

In the figure caption of the copyrighted figure, please include the following text: “Reprinted from [ref] under a CC BY license, with permission from [name of publisher], original copyright [original copyright year].”

b. If you are unable to obtain permission from the original copyright holder to publish these figures under the CC BY 4.0 license or if the copyright holder’s requirements are incompatible with the CC BY 4.0 license, please either i) remove the figure or ii) supply a replacement figure that complies with the CC BY 4.0 license. Please check copyright information on all replacement figures and update the figure caption with source information. If applicable, please specify in the figure caption text when a figure is similar but not identical to the original image and is therefore for illustrative purposes only.

8. Please include captions for your Supporting Information files at the end of your manuscript, and update any in-text citations to match accordingly. Please see our Supporting Information guidelines for more information: http://journals.plos.org/plosone/s/supporting-information.

9. If the reviewer comments include a recommendation to cite specific previously published works, please review and evaluate these publications to determine whether they are relevant and should be cited. There is no requirement to cite these works unless the editor has indicated otherwise.

10.Please review your reference list to ensure that it is complete and correct. If you have cited papers that have been retracted, please include the rationale for doing so in the manuscript text, or remove these references and replace them with relevant current references. Any changes to the reference list should be mentioned in the rebuttal letter that accompanies your revised manuscript. If you need to cite a retracted article, indicate the article’s retracted status in the References list and also include a citation and full reference for the retraction notice.

[Note: HTML markup is below. Please do not edit.]

Reviewers’ comments:

Reviewer’s Responses to Questions

**Comments to the Author**

1. Is the manuscript technically sound, and do the data support the conclusions?

The manuscript must describe a technically sound piece of scientific research with data that supports the conclusions. Experiments must have been conducted rigorously, with appropriate controls, replication, and sample sizes. The conclusions must be drawn appropriately based on the data presented.

Reviewer #1: Partly

Reviewer #2: Partly

**********

2. Has the statistical analysis been performed appropriately and rigorously?

Reviewer #1: N/A

Reviewer #2: N/A

**********

3. Have the authors made all data underlying the findings in their manuscript fully available?

The PLOS Data policy requires authors to make all data underlying the findings described in their manuscript fully available without restriction, with rare exception (please refer to the Data Availability Statement in the manuscript PDF file). The data should be provided as part of the manuscript or its supporting information, or deposited to a public repository. For example, in addition to summary statistics, the data points behind means, medians and variance measures should be available. If there are restrictions on publicly sharing data—e.g. participant privacy or use of data from a third party—those must be specified.

Reviewer #1: Yes

Reviewer #2: Yes

**********

4. Is the manuscript presented in an intelligible fashion and written in standard English?

PLOS ONE does not copyedit accepted manuscripts, so the language in submitted articles must be clear, correct, and unambiguous. Any typographical or grammatical errors should be corrected at revision, so please note any specific errors here.

Reviewer #1: Yes

Reviewer #2: Yes

**********

5. Review Comments to the Author

Please use the space provided to explain your answers to the questions above. You may also include additional comments for the author, including concerns about dual publication, research ethics, or publication ethics. (Please upload your review as an attachment if it exceeds 20,000 characters)

Reviewer #1: This is a very interesting study that makes important contributions to the evidence base for post-diagnostic and peer support for people living with dementia. Below are suggestions to help strengthen the manuscript.

Background and Introduction

This section could be expanded to better situate the study within existing literature and clearly articulate the evidence gap. In particular, further discussion of the extensive peer-support literature in other populations, contrasted with the more limited evidence for people living with dementia, would help readers understand the significance of the study. Additional detail about the Good Life with Dementia course itself, especially its core structure and components, would also be helpful.

Methodology and Methods

Recruitment and consent: More clarity is needed regarding the consent process. Please describe how capacity to provide informed consent was assessed and whether information on participants’ stage of dementia was collected. If individuals living with moderate to severe dementia were excluded, provide a clear rationale. Consider including examples of visual prompts used during recruitment, either in the text or as supplementary material.

Data collection: The manuscript notes that researchers observed all but the first two sessions due to delayed ethical approval. Please discuss how this may have influenced the findings, potentially in the limitations section. Clarify the total number of interviews and focus groups conducted and whether all participants took part in the focus groups. The description of interviews would benefit from further elaboration on the realist interviewing techniques and principles of inclusive dementia research used. When noting that follow-up interviews were conducted “soon after,” please specify the timeframe (e.g., within 1–2 days).

Data Analysis: Please describe how rigor was ensured during the study.

Discussion

Consider integrating literature on similar peer-led courses or programs to further support and strengthen the conclusions. Additionally, the statement on lines 595–596 (“In several of the studies reviewed…”) requires an appropriate citation to substantiate the claim.

Reviewer #2: Thank you for the opportunity to read this paper. Post-diagnostic support for people living with dementia is an important and timely issue. I would recommend addressing the following issues before publication in PLOS One:

- Although the theory of change is integrated throughout the manuscript, there should be more description to ensure readers have sufficient information to understand your approach.

- Methodology and methods: There is a lack of information about the study design. Interviews and focus groups were part of the methods used in this study; however, there is limited information on the kinds of interviews and focus groups that were conducted. At one point, the interviews were described as "realist-informed interviews," but there should be more information to explain what that means in your research. The manuscript would benefit from more information to distinguish the purpose of the interviews compared to the focus groups and what kinds of interview questions and focus group prompts were included (and refined between GL1 and GL2). Any software/coding usage, participant checking, duration and setting of the interview would be some useful information. Additionally, there is a lack of information about the observations and the creation of field notes. Also, one article was cited to support the use of the principles of inclusive dementia research, but this does not provide adequate information on which/how these principles were used in this study. Visual aids were also mentioned (see lines 177-178), but the purpose of these was not clearly explained, such as what these visual aids entailed. Citations should be included to support the sentences about a realist ontology (see lines 118-119) and the epistemological approach that was used (see lines 124-126).

- Supplementary material: The manuscript refers to “Supplementary Document A” (see lines 105 and 233), and two figures were also captioned in the manuscript. It appears that not all of this material was provided. Only one supplementary document titled “S1. Fig initial (pre-research) theory of change” was provided. This S1 document was well designed.

- The analysis of the refined programme theory is strong, particularly for the reflection on the concept of hope. This manuscript also showcased transparency by including the ‘discordant views’ that came up. My recommendation for this section is to clearly explain ‘social shoring-up’ as it came up two more times. The analysis also includes a summary of the consolidated programme theory, which includes themes on shared experience, equality, and positive expectations. However, equality was not well established in relation to the data, and at one point seemed to be conflated with “sameness” (see line 463). I would recommend strengthening the analysis of equality to offer a strong conceptual advance and a robust evaluation in the context of dementia.

Additional note: Under observed outcomes (on line 248), there is an extra period before the sentence "Figure 1" starts.

**********

6. PLOS authors have the option to publish the peer review history of their article (what does this mean?). If published, this will include your full peer review and any attached files.

If you choose “no”, your identity will remain anonymous but your review may still be made public.

**Do you want your identity to be public for this peer review?**  For information about this choice, including consent withdrawal, please see our Privacy Policy.

Reviewer #1: **Yes:**  Adebusola Adekoya

Reviewer #2: No

**********

[NOTE: If reviewer comments were submitted as an attachment file, they will be attached to this email and accessible via the submission site. Please log into your account, locate the manuscript record, and check for the action link "View Attachments". If this link does not appear, there are no attachment files.]

To ensure your figures meet our technical requirements, please review our figure guidelines: https://journals.plos.org/plosone/s/figures

You may also use PLOS’s free figure tool, NAAS, to help you prepare publication quality figures: https://journals.plos.org/plosone/s/figures#loc-tools-for-figure-preparation.

NAAS will assess whether your figures meet our technical requirements by comparing each figure against our figure specifications.

# Author comment: Author response to Decision Letter 1

**DOI:** 10.1371/journal.pone.0349444.r002

21 Apr 2026

These are given in full in the Response to Reviewers letter (in a table - it is hard to reproduce that here, sorry).

Attachment Submitted filename: Response to Reviewers 27th March 2026.docx

# Editor report: Decision Letter 1

**DOI:** 10.1371/journal.pone.0349444.r003

**Authors:** Patricia Anne Morris

1 May 2026

The Good Life with Dementia Approach: A realist-informed qualitative study of a peer-tutored course, co-produced with and for people living with dementia.

PONE-D-25-57625R1

Dear Dr. Gridley,

We’re pleased to inform you that your manuscript has been judged scientifically suitable for publication and will be formally accepted for publication once it meets all outstanding technical requirements.

Within one week, you’ll receive an e-mail detailing the required amendments. When these have been addressed, you’ll receive a formal acceptance letter and your manuscript will be scheduled for publication.

An invoice will be generated when your article is formally accepted. Please note, if your institution has a publishing partnership with PLOS and your article meets the relevant criteria, all or part of your publication costs will be covered. Please make sure your user information is up-to-date by logging into Editorial Manager at Editorial Manager® and clicking the ‘Update My Information’ link at the top of the page. For questions related to billing, please contact billing support.

If your institution or institutions have a press office, please notify them about your upcoming paper to help maximize its impact. If they’ll be preparing press materials, please inform our press team as soon as possible -- no later than 48 hours after receiving the formal acceptance. Your manuscript will remain under strict press embargo until 2 pm Eastern Time on the date of publication. For more information, please contact onepress@plos.org.

Kind regards,

Patricia Anne Morris

Academic Editor

PLOS One

Additional Editor Comments (optional):

Thank you for these thorough revisions. This is exciting research!

Reviewers’ comments:

# Editor report: Acceptance letter

**DOI:** 10.1371/journal.pone.0349444.r004

**Authors:** Patricia Anne Morris

PONE-D-25-57625R1

PLOS One

Dear Dr. Gridley,

I’m pleased to inform you that your manuscript has been deemed suitable for publication in PLOS One. Congratulations! Your manuscript is now being handed over to our production team.

At this stage, our production department will prepare your paper for publication. This includes ensuring the following:

* All references, tables, and figures are properly cited

* All relevant supporting information is included in the manuscript submission,

* There are no issues that prevent the paper from being properly typeset

You will receive further instructions from the production team, including instructions on how to review your proof when it is ready. Please keep in mind that we are working through a large volume of accepted articles, so please give us a few days to review your paper and let you know the next and final steps.

Lastly, if your institution or institutions have a press office, please let them know about your upcoming paper now to help maximize its impact. If they’ll be preparing press materials, please inform our press team within the next 48 hours. Your manuscript will remain under strict press embargo until 2 pm Eastern Time on the date of publication. For more information, please contact onepress@plos.org.

You will receive an invoice from PLOS for your publication fee after your manuscript has reached the completed accept phase. If you receive an email requesting payment before acceptance or for any other service, this may be a phishing scheme. Learn how to identify phishing emails and protect your accounts at https://explore.plos.org/phishing.

If we can help with anything else, please email us at customercare@plos.org.

Thank you for submitting your work to PLOS ONE and supporting open access.

Kind regards,

PLOS ONE Editorial Office Staff

on behalf of

Dr. Patricia Anne Morris

Academic Editor

PLOS One
